# Clustered flexible calibration plots for binary outcomes using random effects modeling

**DOI:** 10.1017/rsm.2025.10046

**Published:** 2025-12-29

**Authors:** Lasai Barreñada, Bavo De Cock Campo, Laure Wynants, Ben Van Calster

**Affiliations:** 1 Department of Development and regeneration, KU Leuven, Belgium; 2 Leuven Unit for Health Technology Assessment Research (LUHTAR), KU Leuven, Belgium; 3 Department of Metabolism, Digestion and Reproduction, Imperial College, United Kingdom; 4 Department of Epidemiology, CAPHRI Care and Public Health Research Institute, Maastricht University, Maastricht, Netherlands; 5 Department of Biomedical Data Sciences, Leiden University Medical Center, Leiden, the Netherlands; 6 Julius Center, Department of Data Science & Biostatistics, University Medical Center (UMC) Utrecht, Utrecht, the Netherlands

**Keywords:** Calibration, prediction models, Clustered Data, Binary Outcomes, Meta-analysis, Model validation

## Abstract

Evaluation of clinical prediction models across multiple clusters, whether centers or datasets, is becoming increasingly common. A comprehensive evaluation includes an assessment of the agreement between the estimated risks and the observed outcomes, also known as calibration. Calibration is of utmost importance for clinical decision making with prediction models, and it often varies between clusters. We present three approaches to take clustering into account when evaluating calibration: (1) clustered group calibration (CG-C), (2) two-stage meta-analysis calibration (2MA-C), and (3) mixed model calibration (MIX-C), which can obtain flexible calibration plots with random effects modeling and provide confidence interval (CI) and prediction interval (PI). As a case example, we externally validate a model to estimate the risk that an ovarian tumor is malignant in multiple centers (*N* = 2489). We also conduct a simulation study and a synthetic data study generated from a true clustered dataset to evaluate the methods. In the simulation study, MIX-C and 2MA-C (splines) gave estimated curves closest to the true overall curve. In the synthetic data study, MIX-C produced cluster-specific curves closest to the truth. Coverage of the PI across the plot was best for 2MA-C with splines. We recommend using 2MA-C with splines to estimate the overall curve and 95% PI and MIX-C for cluster-specific curves, especially when the sample size per cluster is limited. We provide ready-to-use code to construct summary flexible calibration curves, with CI and PI to assess heterogeneity in calibration across datasets or centers.

## Highlights

### What is already known?

Traditional methods for assessing calibration in clinical prediction models often assume independence between observations. However, when performing an external validation where data are clustered, this assumption is violated. Ignoring clustering can impair the reliability of calibration assessments, potentially leading to misguided clinical decisions.

### What is new?

We introduce three novel methodologies that explicitly account for clustering in calibration assessments during clinical prediction model validation. These methods generate an overall calibration curve with PI, representing expected calibration curves in hypothetical new clusters. Among them, the MIX-C method is particularly recommended, as it provides cluster-specific calibration curves, as well as the 2MA-C with splines, as it provides good overall calibration and PI with decent coverage. To facilitate adoption, we provide ready-to-use R functions as part of the *CalibrationCurves* R package.

### Potential impact for RSM readers

External validations aim to assess how clinical prediction models perform across diverse external settings. Neglecting the clustered nature of data in multicenter validations or meta-analyses of validation studies undermines calibration analysis, overlooking cluster-specific insights. The methodologies we propose address these limitations by estimating overall calibration curves and offering PI for hypothetical new clusters. This approach enables more refined and reliable calibration assessments, directly supporting informed decision making in clinical research.

## 1 Introduction

Clinical prediction models (CPMs) are evidence-based tools that estimate the probability of health-related events either at the time of evaluation (diagnosis) or at some point in the future (prognosis).^1^^,^ ^2^ These risk estimates play a crucial role in evidence-based decision making and can be of great value when counseling patients. However, to ensure the reliability of risk estimates, CPMs must exhibit good calibration.^3^ Calibration assesses how well the predicted risks correspond to observed risks.^4^ In recent years, there has been a notable increase in studies involving multiple clusters. The term “cluster” can refer to different groupings within the population under study, depending on the context. For example, in multicenter studies, a cluster commonly refers to different centers, whereas in meta-analysis, a cluster may pertain to each study or to centers within the study where possible (e.g., individual patient data (IPD) meta-analysis^5^). In this work, the term “cluster” will specifically denote a center in a multicenter study, but the methods proposed apply more generally as well. An analysis of CPMs included in Tufts PACE (Predictive Analytics + Comparative Effectiveness), developed after the year 2000 revealed that 64% of the models utilized multicenter (clustered) data, indicating a growing trend in such studies.^6^^,^ ^7^ TRIPOD (Transparent Reporting of a Multivariable Prediction Model for Individual Prognosis or Diagnosis) has addressed this development by publishing an extension known as TRIPOD-Cluster.^8^ This extension underscores the importance of taking clustering into account when developing or evaluating prediction models.

Miscalibration can have a harmful influence on medical decision making.^3^ It is common to observe heterogeneity in model performance across centers or studies, most notably in model calibration.^9^^–^ ^12^ It can thus be misleading to generalize performance results from single-cluster data.^13^^–^ ^16^ For these reasons, it is crucial to collect clustered data to investigate and quantify heterogeneity in calibration performance between clusters. The presence of clustering introduces challenges, as individuals are no longer independent due to correlations among patients within the same cluster.^17^^,^ ^18^ Traditional (non-clustered) methodologies should therefore be adapted for use with clustered data.

The most informative assessment of calibration performance for a prediction model is a calibration plot, which is used to assess whether, among patients with the same estimated risk of the event, the observed proportion of events equals the estimated risk.^4^^,^ ^19^ A calibration plot presents estimated risks on the *x*-axis and the observed proportion on the *y*-axis. For binary outcomes, the observed outcome values are 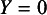 (no event) or 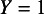 (event). The observed proportion of individuals with the event conditional on the estimated risk is not observed directly, but is estimated. One approach consists of creating 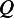 groups (usually based on quantiles of estimated risks) and estimating the observed proportion in each of the groups as the proportion of individuals with the condition. We refer to this approach throughout the text as grouped calibration. The calibration plot then has 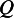 dots representing the mean risk (value on the *x*-axis) and observed proportion (value on the *y*-axis). Another approach is using a flexible model.^20^ The observed outcome is regressed on the estimated probabilities using a smoother such as local regression (LOESS) or splines. The smoothed relation or 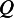 groups are plotted together with a line of identity, which represents perfect calibration. The grouped approach loses information by categorizing the estimated risks and depends on the value of 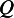 and the grouping approach. Flexible models are dependent on smoothing parameters. Flexible calibration curves have been presented for different problems like survival models^21^, competing risk models^22^, or multiclass models.^23^ Flexible calibration curves that consider clustering have been understudied. Although summarizing statistics of calibration (e.g., O:E) can be meta-analyzed across clusters, such summarizing statistics are by definition less informative than the calibration plot.^19^^,^ ^24^ We present three different approaches to construct flexible calibration curves from clustered data, provide cluster-specific curves, and quantify heterogeneity using prediction intervals (PI). We illustrate the methodologies with a real case example on ovarian cancer data and compare the performance of the methodology using simulated data and synthetic data. Section 2 presents the motivating example, the general notation used throughout the article, and introduces the three approaches to obtain calibration plots accounting for clustering. Sections 3 and 4 present the methodology and results of the simulation study and synthetic data analysis, respectively, and in Section 5, we conclude by discussing our findings. This study is an initial assessment of methods for clustered calibration analysis that we classify as a phase 2 methodological study in the four-phase framework from Heinze et al.^25^

## 2 Methods

### 2.1 Motivating example: ADNEX model and ovarian cancer data

**Figure 1:**
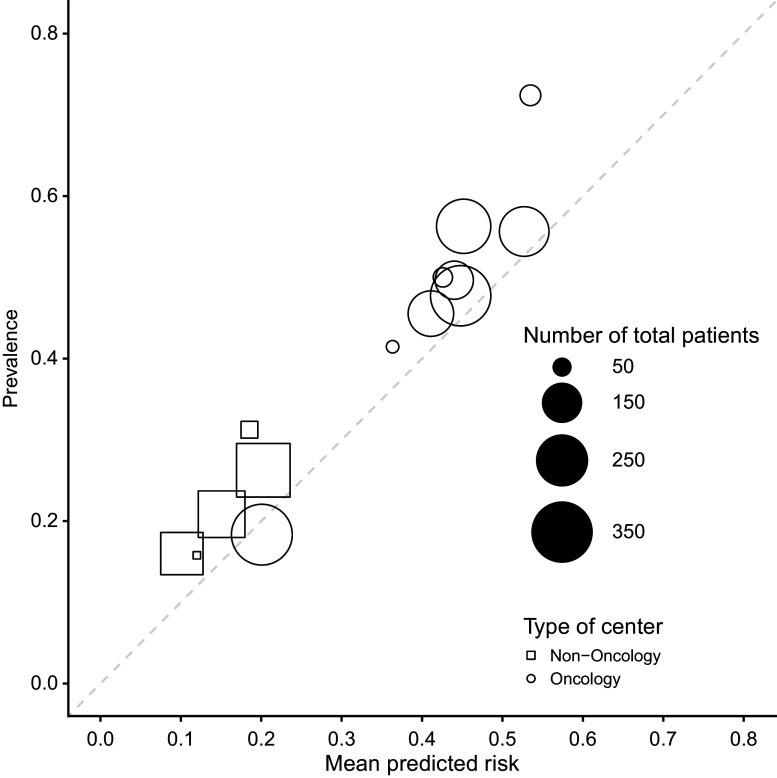
Prevalence and mean predicted ADNEX risk by center across the 14 centers in the dataset. The dashed diagonal line indicates perfect calibration.

The methods will be illustrated using the ADNEX model for ovarian tumor discrimination, which was developed with data from the International Ovarian Tumor Analysis (IOTA) group.^26^^,^ ^27^ The ADNEX model is a multinomial regression mixed model with random intercepts per center to estimate the probability that an ovarian mass is malignant. Predicting the malignancy of ovarian masses prior to surgery is important because benign masses can be managed conservatively, and malignant masses require different surgical approaches depending on the malignant subtype. ADNEX estimates the risk of five outcomes: benign tumor, borderline tumor, stage I invasive cancer, stage II–IV invasive cancer, and secondary metastasis. This work will focus on the overall risk of malignancy, which is obtained by adding the risks for the malignant subtypes. ADNEX has three clinical and six ultrasound predictors: age, serum CA125 level, type of center (oncology center or other hospital type), maximal diameter of the lesion, proportion of solid tissue, number of papillary projections, presence of more than10 locules, acoustic shadows, and ascites. CA125 is optional, and in this work, we focus on ADNEX without CA125. ADNEX coefficients already capture some of the between-center heterogeneity through the type of center. To illustrate multicenter calibration of the ADNEX model, we use an external validation dataset of 2489 patients recruited in 17 hospitals.^28^ Previous external validation of ADNEX on this dataset suggested that there was important heterogeneity between hospitals (Supplementary Figure S1).^9^ To avoid computation errors, three small non-oncology centers in Italy were combined, as well as two small non-oncology centers in the United Kingdom. The dataset then contains 14 clusters, with a median number of 189 patients per cluster (range 38 to 360) (Figure 1). The mean estimated probabilities (range 0.10 to 0.53) and the prevalence of malignancy (range 0.16 to 0.72) varied between clusters. The intra-cluster correlation (ICC) in (logit) malignancy risk based on the null random intercept model was 15%.^17^ An ICC of 0 would mean that all clusters are similar, and all variance in the logit malignancy risk is due to individual-level variability.

### 2.2 Notation

We assume a dataset with a total of 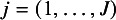 clusters and use 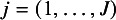 to index the clusters. In each cluster, we have 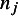 patients, and we index them using 
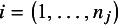
 . The total sample size is 
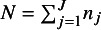
 . We use 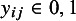 to denote the outcome of patient 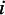 in cluster 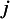 , which takes on the value 0 in the case of a non-event and 1 in the case of an event. We assume that 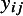 follows a Bernoulli distribution 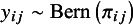 , where 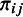 denotes the probability of experiencing the event. Typically, 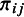 is expressed as a function of a set of risk characteristics, captured in the covariate vector 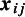 , and we assume that there exists an unknown regression function 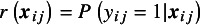 . We approximate this function using a risk prediction model, where we model the outcome as a function of the observed risk characteristics and employ statistical or machine learning techniques to estimate this model. A general expression that encompasses both types is(1)

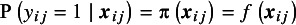

where 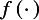 denotes the predicted risk given covariate vector 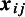 . In a logistic regression model, we have that

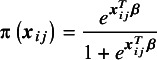

 where 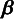 denotes the parameter vector. We can rewrite the above equation as

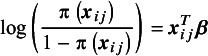




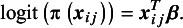



We can employ splines to allow for a non-linear relationship between the covariates and the outcome.^29^ To estimate equation (1), we fit a statistical or machine learning model to the training data. The resulting model fit then provides us with the predicted probability 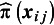 .

Using calibration curves, we assess how well the predicted probabilities correspond to the actual event probabilities.^1^^,^ ^4^^,^ ^30^^,^ ^31^ A calibration plot maps the predicted probabilities 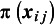 to the actual event probabilities 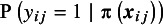 and hereby provides a visual representation of the alignment between the model’s estimated risks and the true probabilities. For a perfectly calibrated model, the calibration curve follows the diagonal as 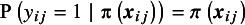 for all 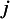 and all 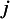 .

### 2.3 Flexible calibration plots ignoring clustering

We can estimate the calibration curve using a logistic regression model^31^^–^ ^33^


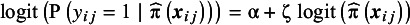

 where we estimate the observed proportions as a function of the (logit-transformed) predicted probabilities. Using the fitted model, we create a calibration curve by plotting 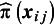 against the observed proportions 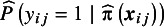 . This model, however, only allows for a linear relationship and, as such, does not adequately capture moderate calibration. To allow for a non-linear relationship between 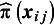 and 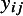 , we can rely on non-parametric smoothers, such as locally estimated scatterplot smoothing (LOESS) or restricted cubic splines

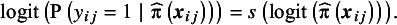



Here, 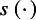 denotes the smooth function applied to the logit-transformed predicted probability. This results in a flexible calibration plot, which is implemented in R packages such as *CalibrationCurves* (val.prob.ci function), *rms* (val.prob function), or *tidyverse* (cal_plot_logistic function).^4^^(p. 20),^ ^29^^,^ ^34^^,^ ^35^

**Figure 2:**
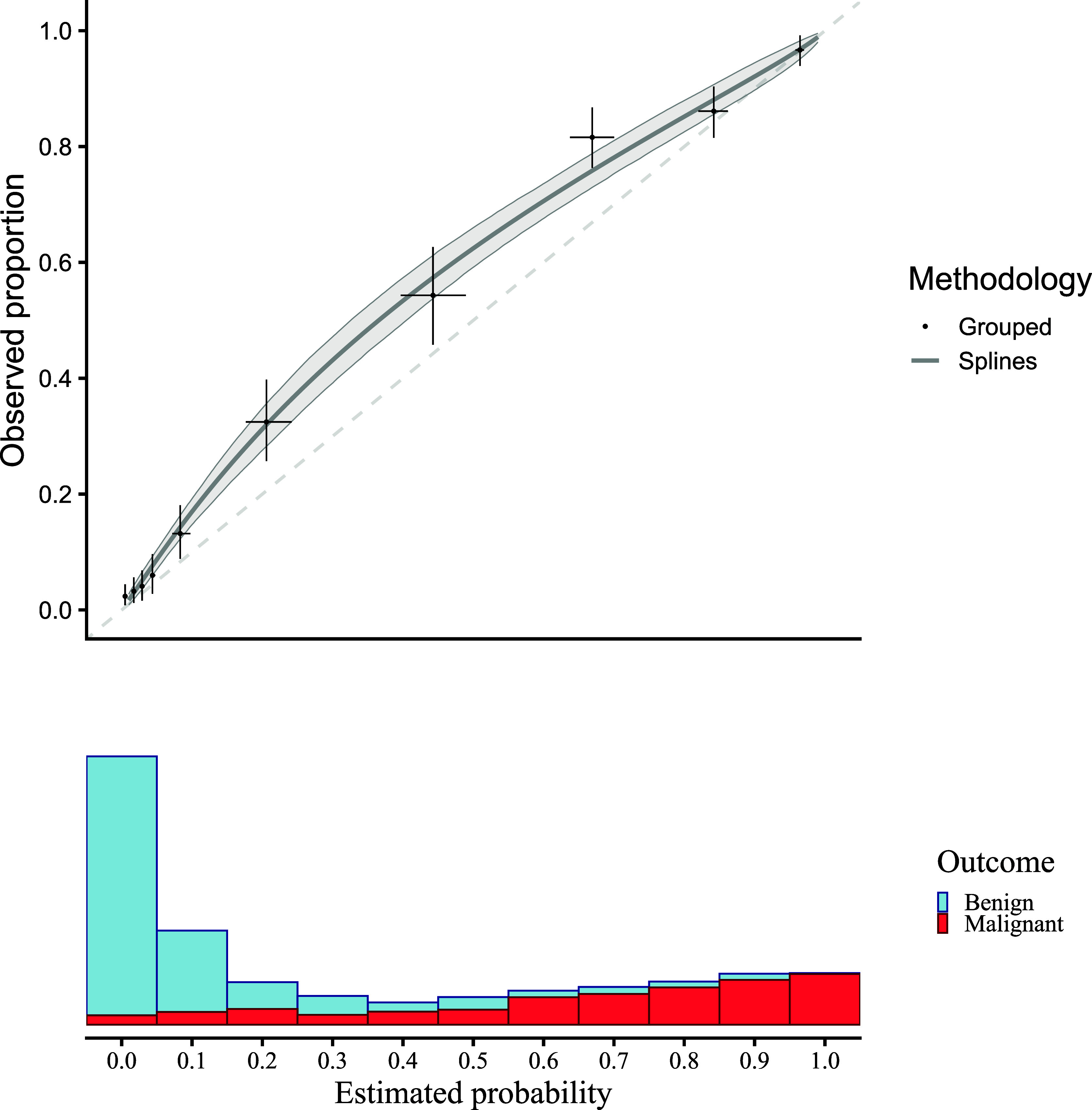
Traditional flexible calibration curves for the ADNEX model in the motivating example. Observed proportion is estimated with a logistic model with restricted cubic splines to model non-linear effects, and estimated risks are grouped in 10 groups. Confidence intervals are shown for 1000 bootstraps with a shaded area for splines and a + for grouped calibration. The dashed diagonal line indicates perfect calibration.

Figure 2 presents a flexible calibration curve (estimated using a restricted cubic spline) based on the motivating example with pooled data, and the results of a grouped calibration assessment for ovarian malignancy prediction. The panel below shows the sample distribution of the estimated risks for benign cases (blue) and malignant cases (red). The plots suggest that risks were estimated too low.

Since there is no commonly accepted way to estimate calibration curves for prediction models in clustered data, we present three approaches covering different statistical methodologies. First, we present a grouped clustered calibration plot based on a bivariate random effects meta-analysis model (clustered group calibration, CG-C). Second, we introduce a two-stage univariate random effects meta-analytical approach for the estimation of the observed events (two-stage meta-analysis calibration, 2MA-C). Finally, we provide a one-step approach where we fit a random effects model with smooth effects to obtain individual calibration slopes per cluster (mixed model calibration, MIX-C). We present the summary of the three proposed approaches in Table 1.

**Table 1:** Overview of introduced methodologies for creating flexible calibration curves accounting for clustering

Method	Estimation of observed proportion	Strengths	Limitations	Implementation	Illustration
**CG-C**	**Grouped**: Bivariate random effects meta-analysis of logit-transformed mean estimated risk and event rate by quantile per cluster **Interval**: Bivariate random effects meta-analysis of logit-transformed mean estimated risk and event rate by estimated risk interval	Model agnostic Pointwise CI and PI All clusters have the same number of groups	Computation time Groups can contain observations with very different estimated risks within and between clusters (grouped version) Clusters may not have the same number of groups (e.g., risk intervals without observations) (interval version) Curves depend on the number of groups	CGC(method = “grouped”) CGC(method = “interval”)	Supplementary Figures S2–S3 Supplementary Figures S4–S5
**2MA-C**	Random effects meta-analysis of estimated smooth observed proportion by cluster **Splines**: Recommended when clusters are small **LOESS**: More flexible but can fail with small clusters	Pointwise CI and PI	Computation time Curve depends on the smoother used in the cluster-specific models	MAC2(method_choice = “splines”) MAC2(method_choice = “loess”)	Supplementary Figure S6 (splines) Supplementary Figure S7 (LOESS)
**MIX-C**	Logistic generalized linear mixed model with restricted cubic splines	Curve-wise CI and PI Also provides shrunken curves per center	Computation time	MIXC(model = “slope”)	Supplementary Figure S8 (slope)

*Note:* Implementation in the code included in the OSF. For the implementation in *CalibrationCurves* package go to the package documentation.

### 2.4 Clustered group calibration (CG-C)

The CG-C is a two-stage approach that extends the traditional grouped calibration to clustered data. In the traditional approach, data are pooled and groups are created based on quantiles (often deciles). In CG-C, we create 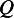 quantiles in every cluster (based on the estimated risk distribution per cluster) with 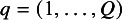 . Hereafter, we pool the estimated risks and observed proportions with a bivariate random effects meta-analysis at each quantile 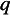 . The logit-transformed observed proportion and average estimated risk are the response variables, and we capture the cluster-specific deviation by including a random intercept. The equation of this model is given by(2)

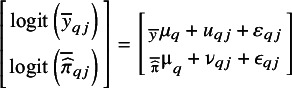


where 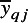 denotes the prevalence of cluster 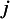 within quantile 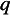 and 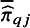 the average estimated risk in cluster 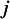 within quantile 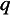 . We use the subscript 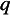 in the quantities to indicate that this refers to quantile 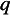 . 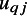 and 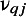 represent the random intercepts for cluster 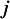 , capturing the between-cluster heterogeneity, whereas 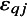 and 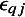 account for the within-cluster error. In this model, we assume that 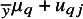 and 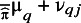 are randomly drawn from a distribution with mean (or pooled) 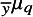 and 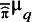 , respectively. Additionally, we assume that

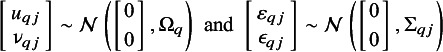

 where 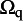 and 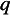 represent the between-cluster and within-cluster covariance matrices within quantile 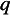 , respectively

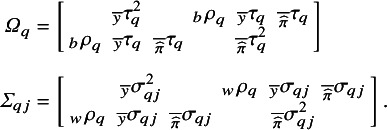



We use 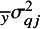 and 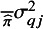 to denote the variance of 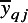 and, 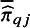 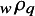 represents the within-cluster correlation and 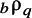 the between-cluster correlation in quantile *q*. The between-cluster variance is denoted as 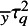 and 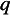 . For each quantile 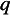 , we use a bivariate meta-analysis model to account for the strong dependence between the average estimated risk and observed proportion. The calibration plot is then created by plotting the 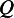 values of 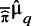 on the *x*-axis and 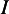 on the *y*-axis. This approach has the advantage of obtaining an observed proportion per cluster in a model-agnostic way. In addition, it provides uncertainty measures in the cluster with the average random effect with confidence intervals (CI) for that effect and in a hypothetical new cluster (PI). It also has some limitations. First, it has the same drawback as the traditional grouped calibration because the curve is dependent on the number of quantiles selected, especially when the sample size is limited. Second, and linked to the previous limitation, the arbitrary selection of 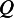 quantiles might create groups that are too heterogeneous between clusters because the estimated risk distributions differ (e.g., a high-risk setting vs a low-risk setting). Third, the meta-analysis does not provide cluster-specific curves using empirical Bayes. We used the *rma.mv* function of the *metafor* package.^36^

We include an extension to the proposed methodology that we call “interval” grouping. The methodology is the same except for the way the groups are created. Per cluster, we create 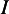 groups by dividing the probability space (0–1) into 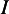 equally spaced intervals. This way, we will create 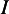 groups of different sizes based on the estimated risks. By doing this, we reduce the within-group variability at the expense of not necessarily having all clusters present in all 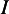 groups. The algorithm, additional details on the implementation, and illustration are available in the Supplementary Material (Appendix A1, Figures S2–S5), and code for the implementation in the OSF repository.^37^

### 2.5 Two-stage meta-analysis calibration (2MA-C)

For the second approach, we use a two-stage random effects meta-analysis to estimate the calibration plot. First, we fit a flexible curve per cluster with a smoother of choice. Currently, we have implemented restricted cubic splines and LOESS. For LOESS, in each cluster, the span parameter with the lowest bias-corrected AIC is selected. For restricted cubic splines, the number of knots per cluster is selected by performing a likelihood ratio test between all combinations of models with three, four, and five knots, selecting the model with the fewest knots that provides the best fit. We train a flexible curve per cluster and estimate the observed proportion for a fixed grid of estimated risks from 0.01 to 0.99 ( 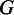 , default is 100 points). Hereafter, we use a random effects meta-analysis model per point in the grid to combine the logit-transformed predictions across clusters, fitting the following univariate model per point 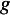 in the grid

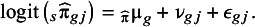




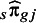
 denotes the predicted proportion of point 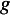 for cluster 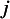 , 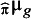 the overall mean for point 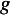 , 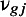 the cluster-specific deviation, and 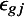 the error. We assume that 
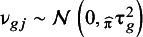
 and 
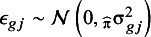
 . Furthermore, we include the inverse of the variance of 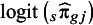 as weight and thus take both the cluster-specific ( 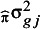 ) and between-cluster variability ( 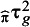 ) into account. As such, clusters with more precise estimates are assigned greater weights. This approach has the strength of being easy to compute and providing heterogeneity measures for the cluster with average calibration. This allows us to plot CI and PI, which help visually assess the certainty of the average curve (CI) and the heterogeneity between hypothetical new clusters (PI) in the whole range of predicted probabilities. The main limitation is that it is based on the smoothing technique used in each individual cluster, so the curves will vary depending on the smoother selected. Additionally, the technique treats each point in the grid as independent, leading to pointwise CI and PI. Cluster-specific curves are obtained in the first stage independently of the rest of the clusters. The algorithm, additional details on the implementation, and illustration are available in the Supplementary Material (Appendix A2, Figures S6–S7), and code for the implementation in the OSF repository.

### 2.6 Mixed model calibration (MIX-C)

In the third approach, we employ a one-stage logistic generalized linear mixed model (GLMM) to model the outcome as a function of the logit-transformed predictions. To allow for a non-linear effect, we employ restricted cubic splines with three knots for both the fixed and random effects

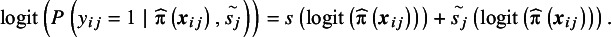



Here, 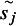 denotes the smooth random effect for cluster 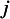 . The model is fit using *lme4* ^38^ and splines are added with *rms* package.^39^ This approach estimates the calibration per cluster and the variance of the random effects in a single step. As opposed to the random effects in 2MA-C, the MIX-C model takes all observations across the entire spectrum of predicted risk into account when predicting the realized values of the random effects and cluster-specific calibration curves. The main limitation is the computation time needed when the number of clusters is large. The algorithm, additional details on the implementation, and illustration are available in the Supplementary Material (Appendix A3, Figure S8), and code for the implementation is available in the OSF repository.

### 2.7 Results for the case example

**Figure 3:**
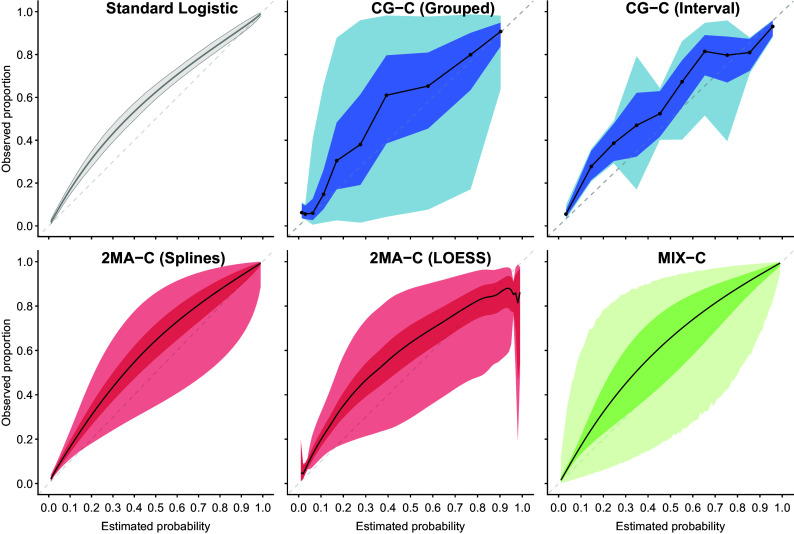
Comparison of the standard logistic regression with splines and the three introduced methodologies with CI (bright shaded) and PI (light shaded). Number of quantiles for CG-C were 10, 2MA-C fitted center-specific curves with splines or LOESS, and MIX-C used random intercept and slopes with restricted cubic splines and three knots. The dashed diagonal line indicates perfect calibration.

A visual analysis of the different curves shows that all the approaches present similar results on the case example, in line with the previously obtained results ignoring clustering (Figure 3). All plots suggest that ADNEX underestimated risks. Nevertheless, there are important differences in the estimated uncertainty and heterogeneity. Details, visualizations, and variations of each method are shown in Supplementary Figures S2–S8. The methods are available in the *CalibrationCurves* package.^45^

## 3 Simulation study

The motivating example showcases the applicability of the methods. However, with real data, it is not possible to know the true underlying observed proportion. Therefore, we designed a simulation study to explore the performance of the introduced approaches and compare it with cluster ignorant methodologies.

### 3.1 Methods

The data-generating models were based on logistic regression with a random intercept per cluster; hence, the formula to obtain the true probabilities was of the form 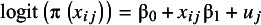 . 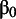 represents the intercept, 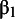 is the corresponding effect of covariate 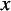 , and 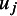 is the cluster-specific deviation. We first obtained the true models according to a full factorial design where two factors were varied: little vs strong clustering (ICC 5% or 20%) and lower vs higher true area under the receiver operating characteristic curve (AUC;0.75 and 0.9). Event rate was fixed at 30% for the whole population but varied between clusters according to the variance of the random intercept 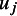 . For each data-generating mechanism, we generated data for 200 clusters and 2,000,000 patients (10,000 observations per cluster) and a single normally distributed linear predictor ( 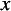 , which can be seen as a combination of several predictors) with mean 0 and variance 1. Random effects 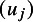 were also normally distributed with variance according to the desired ICC. ICC is calculated as the variance of a null random intercept model divided by the total variance, calculated as the sum of the variances of the null random intercept model and the standard logistic distribution.^40^ AUC was controlled by varying the coefficient ( 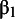 ), and the desired event rate was controlled by modifying the intercept 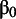 . We obtained four superpopulations by trial and error (Supplementary Table S1). The code to obtain the superpopulations is available in the OSF repository.

In each of the superpopulations, we drew four scenarios, inspired by real validation or development studies, varying events per cluster (EPC) (20 vs 200) and number of clusters (5 vs 30). These scenarios refer to the dataset on which hypothetical prediction models are developed or validated. This results in 16 scenarios overall, by combining all values for ICC, AUC, EPC, and number of clusters. We then applied the methods for varying development or varying validation sample size.

#### 3.1.1 Varying development size

The clusters and patients within each cluster were selected randomly, and the number of patients was selected according to the prevalence and the desired EPC ( 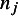 = EPC * 1.15 divided by cluster prevalence; we multiplied EPC to ensure that the desired EPC is obtained across all clusters). We developed logistic regression models with restricted cubic splines and three knots in these training datasets. Then, we externally validated the calibration of the model in patients from clusters not used for model development in an ideal situation with a high number of clusters and high EPC. Therefore, we validated models on a random selection of 100,000 observations from a random selection of 30 clusters that were not sampled for model development. On average, each cluster contained 1,000 events and 3,333 observations, but the number of events varied depending on the cluster-specific event rate.

#### 3.1.2 Varying validation size

Additionally, we validated the models with varying validation sample sizes. In this case, we fixed the model to be validated to a logistic regression model with restricted cubic splines and three knots trained with a sufficiently large sample size according to Riley’s criteria (*N* = 1711) from a cluster with average event rate in each of the superpopulations.^41^ Then, we validated those models varying EPC (20 vs 200) and the number of clusters (5 vs 30) in the validation sample.

We applied the CG-C (grouped and interval), 2MA-C (splines and LOESS), and MIX-C approaches to the validation sample varying development and validation sample size. For comparison, we also obtained a standard flexible logistic calibration model (i.e., ignoring clustering, see Section 2.3) using restricted cubic splines and three knots. True risks are obtained using the formula in Supplementary Table S1, and the true risks in the cluster with the average effect are obtained by setting the random intercept to 0. These true risks can be used to generate true calibration curves per cluster and for the cluster with the average effect. To numerically compare the deviation of the estimated calibration curve from the true one, we calculated the mean squared calibration error (MSCE) as the mean squared difference between the estimated observed proportions and the true observed proportions (based on the true risks) in the cluster with the average effect (setting 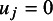 ) over a fixed grid of 100 estimated risks (100 evenly spaced points from 0.01 to 0.99).^23^ For CG-C, the grid contains 10 points by definition. The process was repeated 100 times per scenario.

#### 3.1.3 Heterogeneity

We evaluated the coverage of 95% PI. For each of the three methods, using the same grid of values that we used for the MSCE, we evaluated whether the true cluster-specific risk (including random effects) fell within the PI at each of the grid points. This means that we obtained the true cluster-specific probabilities using the formula in Supplementary Table S1 and compared for each clustered calibration approach whether the PI contained the true values. Coverage was calculated as the proportion over the 200 clusters in the superpopulations.

### 3.2 Results

**Figure 4:**
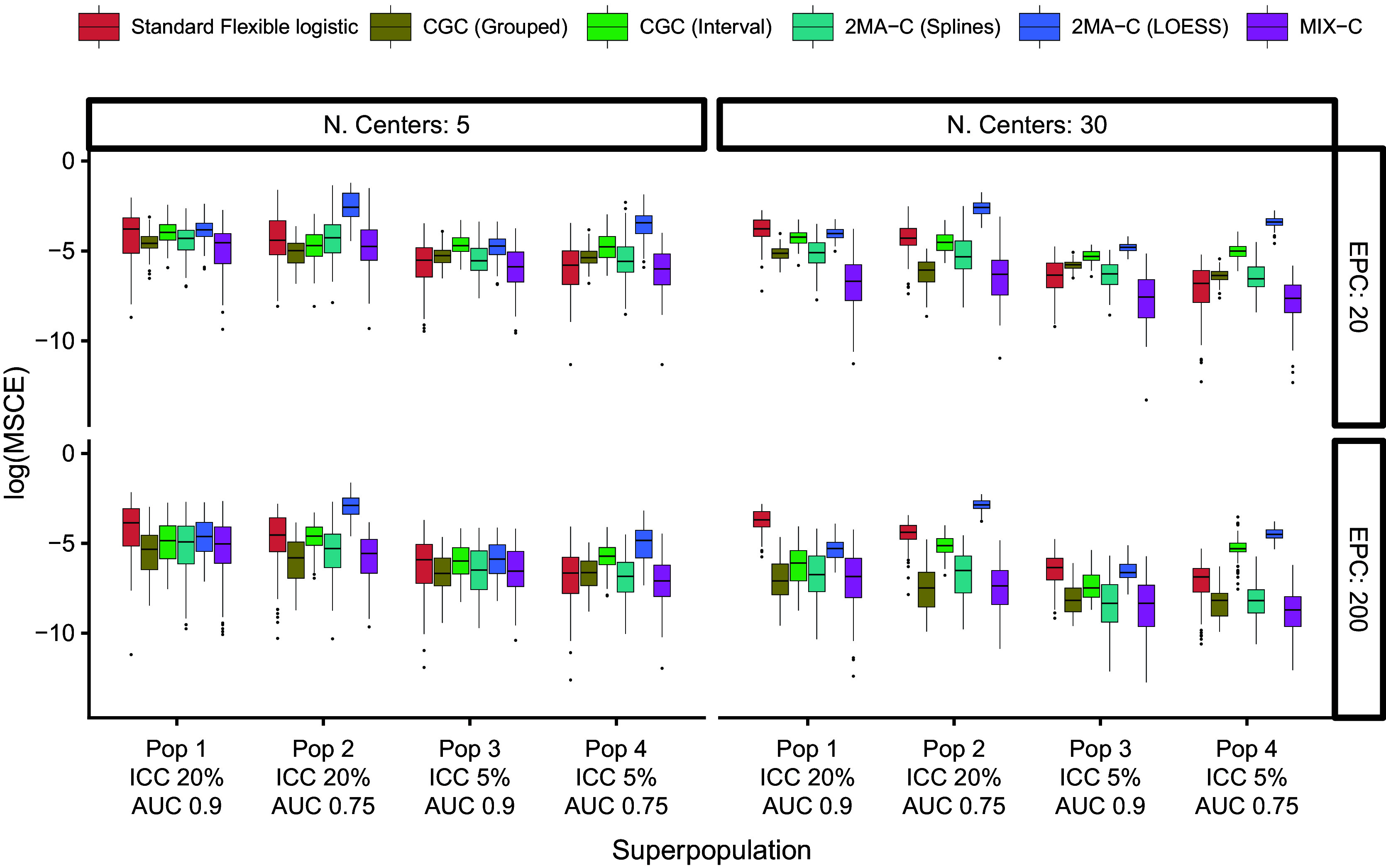
Boxplots of mean squared calibration error (log) for fixed prediction models varying validation events per center (EPC) and the number of centers in the validation.

The median MSCE results for varying validation sample sizes are displayed in Table 2 (multiplied by 100) and Figure 4. Note that CG-C focuses on 10 points, whereas the other approaches focus on a grid of 100 points, so CG-C and the other approaches are not directly comparable. MIX-C was the best performing approach in all cases. 2MA-C (splines) also performed well in scenarios with high validation sample size. In general, the MSCE was better with high EPC, low ICC, and more clusters in the validation. Standard flexible logistic calibration performed considerably worse in all scenarios, in particular when ICC was high. When evaluated on a large validation sample with varying development size, the results also showed that 2MA-C (splines) and MIX-C were the best methods across all scenarios (Table 3 and Supplementary Figure S9).

**Table 2:** Median (IQR) squared difference between true average probabilities and estimated observed proportion (MSCE) with logistic calibration, CG-C (10 groups), 2MA-C, and MIX-C methods for a logistic model varying validation events per center and number of centers. Bold numbers indicate best performance across approaches

EPC	N. Cent	ICC	AUC	Standard flexible logistic	CG-C group^a^	CG-C interval^a^	2MA-C splines	2MA-C LOESS	MIX-C
20	5	0.05	0.75	0.31 (0.10–0.68)	0.46 (0.34–0.66)	0.85 (0.47–1.51)	0.38 (0.21–0.85)	3.26 (1.78–4.79)	**0.25 (0.10–0.57)**
20	5	0.05	0.9	0.40 (0.16–0.81)	0.52 (0.35–0.70)	0.90 (0.66–1.42)	0.39 (0.23–0.78)	0.89 (0.55–1.32)	**0.28 (0.12–0.64)**
20	5	0.2	0.75	1.22 (0.55–3.63)	0.69 (0.35–1.04)	0.91 (0.50–1.67)	1.40 (0.61–2.91)	7.67 (4.59–16.77)	**0.87 (0.40–2.18)**
20	5	0.2	0.9	2.29 (0.60–4.26)	1.03 (0.79–1.51)	1.89 (1.24–2.94)	1.36 (0.72–2.15)	2.20 (1.52–3.17)	**1.07 (0.33–1.77)**
20	30	0.05	0.75	0.11 (0.04–0.23)	0.17 (0.14–0.22)	0.67 (0.50–0.87)	0.14 (0.09–0.28)	3.36 (2.82–4.08)	**0.05 (0.02–0.10)**
20	30	0.05	0.9	0.18 (0.09–0.35)	0.31 (0.26–0.36)	0.50 (0.40–0.65)	0.19 (0.10–0.32)	0.82 (0.69–0.97)	**0.05 (0.02–0.14)**
20	30	0.2	0.75	1.38 (0.93–2.37)	0.23 (0.12–0.36)	1.09 (0.69–1.65)	0.49 (0.25–1.18)	7.59 (5.36–9.76)	**0.18 (0.06–0.40)**
20	30	0.2	0.9	2.32 (1.53–3.76)	0.59 (0.45–0.76)	1.46 (1.05–1.85)	0.62 (0.35–1.07)	1.78 (1.41–2.22)	**0.13 (0.04–0.31)**
200	5	0.05	0.75	0.13 (0.04–0.31)	0.13 (0.06–0.25)	0.33 (0.21–0.54)	0.11 (0.05–0.23)	0.79 (0.30–1.40)	**0.08 (0.04–0.20)**
200	5	0.05	0.9	0.27 (0.07–0.64)	0.13 (0.06–0.30)	0.25 (0.12–0.53)	**0.15 (0.05–0.44)**	0.28 (0.13–0.62)	**0.15 (0.06–0.43)**
200	5	0.2	0.75	1.07 (0.43–2.80)	0.30 (0.10–0.73)	1.01 (0.60–1.67)	0.51 (0.17–1.13)	5.56 (3.41–8.37)	**0.39 (0.13–0.84)**
200	5	0.2	0.9	2.10 (0.58–4.67)	0.49 (0.16–1.06)	0.78 (0.29–1.79)	0.73 (0.22–1.76)	0.98 (0.43–2.16)	**0.66 (0.22–1.69)**
200	30	0.05	0.75	0.10 (0.04–0.17)	0.03 (0.01–0.04)	0.50 (0.42–0.69)	0.03 (0.01–0.05)	1.12 (0.91–1.43)	**0.02 (0.01–0.03)**
200	30	0.05	0.9	0.18 (0.09–0.29)	0.03 (0.02–0.06)	0.06 (0.03–0.12)	**0.02 (0.01–0.07)**	0.13 (0.10–0.21)	**0.02 (0.01–0.07)**
200	30	0.2	0.75	1.25 (0.85–1.83)	0.06 (0.02–0.14)	0.59 (0.41–0.88)	0.15 (0.04–0.33)	5.73 (4.70–7.18)	**0.06 (0.02–0.15)**
200	30	0.2	0.75	2.50 (1.68–3.97)	0.08 (0.04–0.21)	0.23 (0.08–0.45)	0.12 (0.05–0.34)	0.51 (0.31–0.72)	**0.11 (0.03–0.29)**

*Note*: MSCE is multiplied by 100. The lower the number, the closer the estimated summary calibration curve is to the true calibration curve in a cluster with an average effect. Rounded to two decimals.In bold are the best working approach(es) for estimating observed proportions excluding CG-C methods.

Abbreviations: EPC: Event per center in the **validation** sample; N.Cent: Number of centers in the **validation** sample.

a Deciles is calculated only with 10 points instead of 100.

**Table 3:** Median (IQR) squared difference between true average probabilities and estimated observed proportion (MSCE) with logistic calibration, CG-C (10 groups), 2MA-C, and MIX-C methods for a logistic model varying training sample events per center and number of centers. Bold numbers indicate best performance across approaches

EPC	N. Cent	ICC	AUC	Standard flexible logistic	CG-C group^a^	CG-C interval^a^	2MA-C splines	2MA-C LOESS	MIX-C
20	5	0.05	0.75	0.03 (0.02–0.06)	0.01 (0.00–0.03)	0.51 (0.27–0.70)	**0.01 (0.00–0.03)**	0.54 (0.16–1.45)	0.02 (0.01–0.04)
20	5	0.05	0.9	0.05 (0.04–0.09)	0.02 (0.01–0.04)	0.03 (0.01–0.06)	**0.03 (0.01–0.05)**	0.06 (0.03–0.10)	**0.03 (0.01–0.06)**
20	5	0.2	0.75	0.27 (0.13–0.80)	0.06 (0.02–0.16)	1.13 (0.56–2.07)	**0.06 (0.02–0.17)**	4.54 (3.13–8.44)	0.12 (0.03–0.61)
20	5	0.2	0.9	0.36 (0.27–0.46)	0.05 (0.02–0.18)	0.17 (0.06–0.36)	**0.06 (0.01–0.20)**	0.46 (0.29–0.87)	**0.06 (0.02–0.20)**
20	30	0.05	0.75	**0.02 (0.01–0.04)**	0.02 (0.00–0.04)	0.47 (0.33–0.59)	**0.02 (0.00–0.03)**	0.40 (0.20–0.72)	**0.02 (0.00–0.03)**
20	30	0.05	0.9	0.05 (0.03–0.09)	0.02 (0.01–0.04)	0.02 (0.01–0.07)	**0.02 (0.01–0.07)**	0.05 (0.03–0.09)	**0.02 (0.01–0.06)**
20	30	0.2	0.75	0.16 (0.10–0.24)	0.04 (0.01–0.14)	1.15 (0.72–1.75)	**0.04 (0.02–0.11)**	6.06 (4.61–8.15)	0.05 (0.02–0.13)
20	30	0.2	0.9	0.36 (0.27–0.44)	0.06 (0.02–0.15)	0.18 (0.08–0.32)	**0.06 (0.02–0.17)**	0.63 (0.46–0.91)	**0.06 (0.02–0.16)**
200	5	0.05	0.75	0.02 (0.01–0.03)	0.01 (0.00–0.03)	0.42 (0.34–0.58)	**0.01 (0.00–0.02)**	0.41 (0.24–0.61)	**0.01 (0.00–0.02)**
200	5	0.05	0.9	0.05 (0.03–0.08)	0.02 (0.01–0.04)	0.03 (0.01–0.06)	**0.02 (0.01–0.05)**	0.05 (0.03–0.10)	**0.02 (0.01–0.05)**
200	5	0.2	0.75	0.14 (0.10–0.21)	0.05 (0.02–0.13)	1.23 (0.64–2.03)	**0.05 (0.02–0.11)**	6.55 (4.45–7.90)	**0.05 (0.02–0.11)**
200	5	0.2	0.9	0.34 (0.24–0.46)	0.06 (0.03–0.18)	0.13 (0.06–0.36)	**0.06 (0.03–0.21)**	0.46 (0.26–0.90)	**0.06 (0.03–0.19)**
200	30	0.05	0.75	0.02 (0.01–0.03)	0.01 (0.00–0.03)	0.44 (0.38–0.54)	**0.01 (0.00–0.03)**	0.41 (0.22–0.68)	**0.01 (0.00–0.03)**
200	30	0.05	0.9	0.05 (0.03–0.08)	0.02 (0.00–0.04)	0.02 (0.01–0.07)	**0.02 (0.01–0.07)**	0.04 (0.03–0.10)	**0.02 (0.00–0.07)**
200	30	0.2	0.75	0.13 (0.09–0.19)	0.05 (0.02–0.14)	1.17 (0.62–2.05)	0.05 (0.02–0.12)	6.71 (5.57–7.72)	**0.04 (0.02–0.10)**
200	30	0.2	0.9	0.31 (0.24–0.51)	0.07 (0.02–0.18)	0.16 (0.09–0.32)	0.08 (0.01–0.20)	0.62 (0.46–0.85)	**0.07 (0.01–0.19)**

*Note*: MSCE is multiplied by 100. The lower the number, the closer the estimated summary calibration curve is to the true calibration curve in a cluster with an average effect. Rounded to two decimals. In bold are the best working approach(es) for estimating observed proportions excluding CG-C methods.

EPC: Event per center in the **training** sample; N.Cent: Number of centers in the **training** sample.

a Deciles is calculated only with 10 points instead of 100.

**Figure 5:**
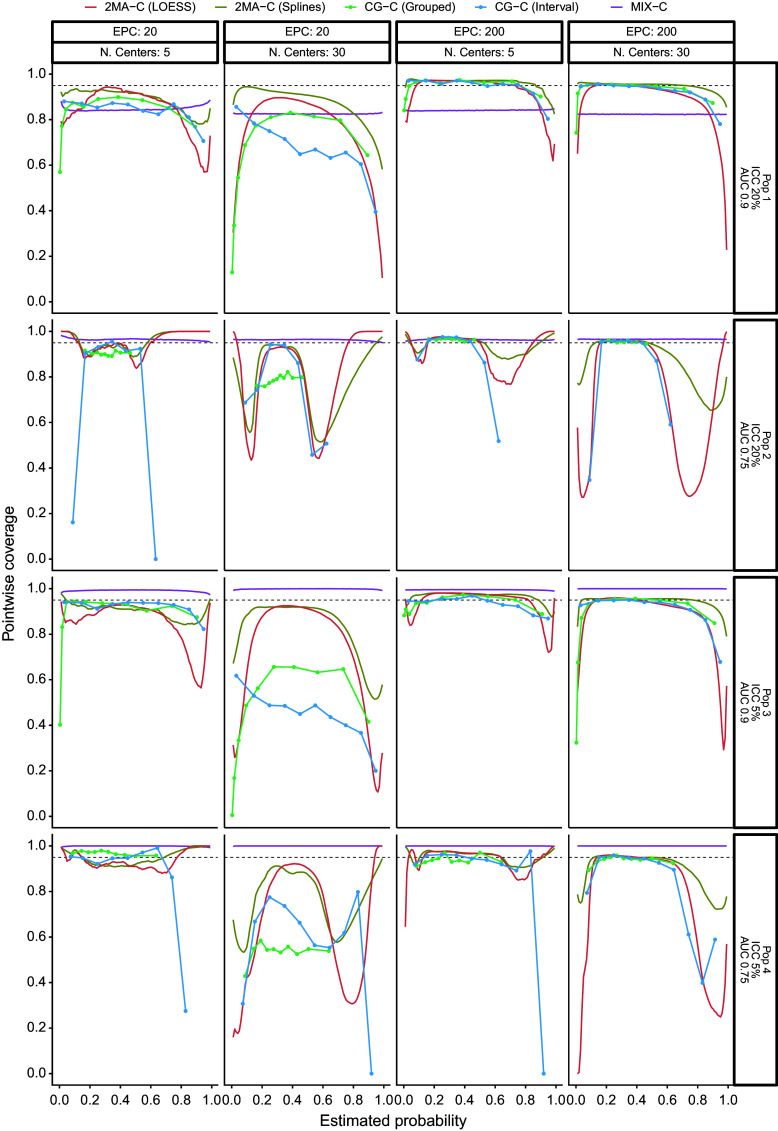
Pointwise prediction interval coverage varying the validation sample size. The model validated is the same in each superpopulation, and it was trained from a center with average event rate and with adequate sample size. The black dotted line indicates nominal coverage (95%).

The PI coverage with varying validation sample sizes was close to nominal with high EPC and low ICC and when using the 2MA-C (splines) approach (Figure 5). When EPC was high, the coverage was close to nominal in the region where validation data were available but very poor at the tails (i.e., regions with few validation data) except for the MIX-C approach. MIX-C had correct coverage when ICC was high and AUC was low, too wide PI when ICC was low, and too narrow PI when ICC and AUC were high. When EPC was low, increasing the number of clusters reduced the coverage notably, especially for the grouped approaches and at the tails. The coverage in the large validation dataset was poor at the tails except for MIX-C (Supplementary Figure S10). High AUC and low ICC in development tended to have better PI coverage. Code and data to reproduce the simulation study and analysis are available in the OSF repository.

## 4 Synthetic data

The simulation study showed that the methods correctly estimate observed proportions under different logistic DGMs. In the real world, the link between outcome and predictors is often not perfectly defined by a linear or logistic function. Therefore, we aimed to test the methods in a more flexible situation using a synthetically generated dataset based on the data used in the motivating example.

### 4.1 Methods

To generate synthetic data, we used data from the International Ovarian Tumor Analysis (IOTA) consortium that was used to develop prediction models to estimate the risk of malignancy in patients with an ovarian tumor.^9^^,^ ^26^ We used the *synthpop* package in R, which learns the structure of the data and generates a new dataset where IPD are masked, but the underlying structure is preserved.^42^ We generated synthetic data for 1 million individuals for each of the 10 hospitals separately and used these synthetic patients as cluster-specific populations (retaining the clustered structure of the original dataset). We generated two true models per center: one based on a logistic regression model with splines for continuous variables and the other based on a random forest model with the number of randomly selected parameters per split (mtry) set to three and the depth of the individual trees set to 10 (minimum node size).^43^ Both models were trained on the real data from that center, using the nine ADNEX predictors listed above, excluding type of center. The true models were applied to the 1 million synthetic patients, and the outcome was generated with Bernoulli trials based on the true risks of malignancy from the applied models. This means that the same synthetic patient has two true risks and can have two different outcomes. The code to generate the synthetic data is available in the OSF repository, but the original data or the synthetic data are not publicly available. The comparison of the real and synthetic data for quality check in one center is presented in Supplementary Figure S11.

**Figure 6:**
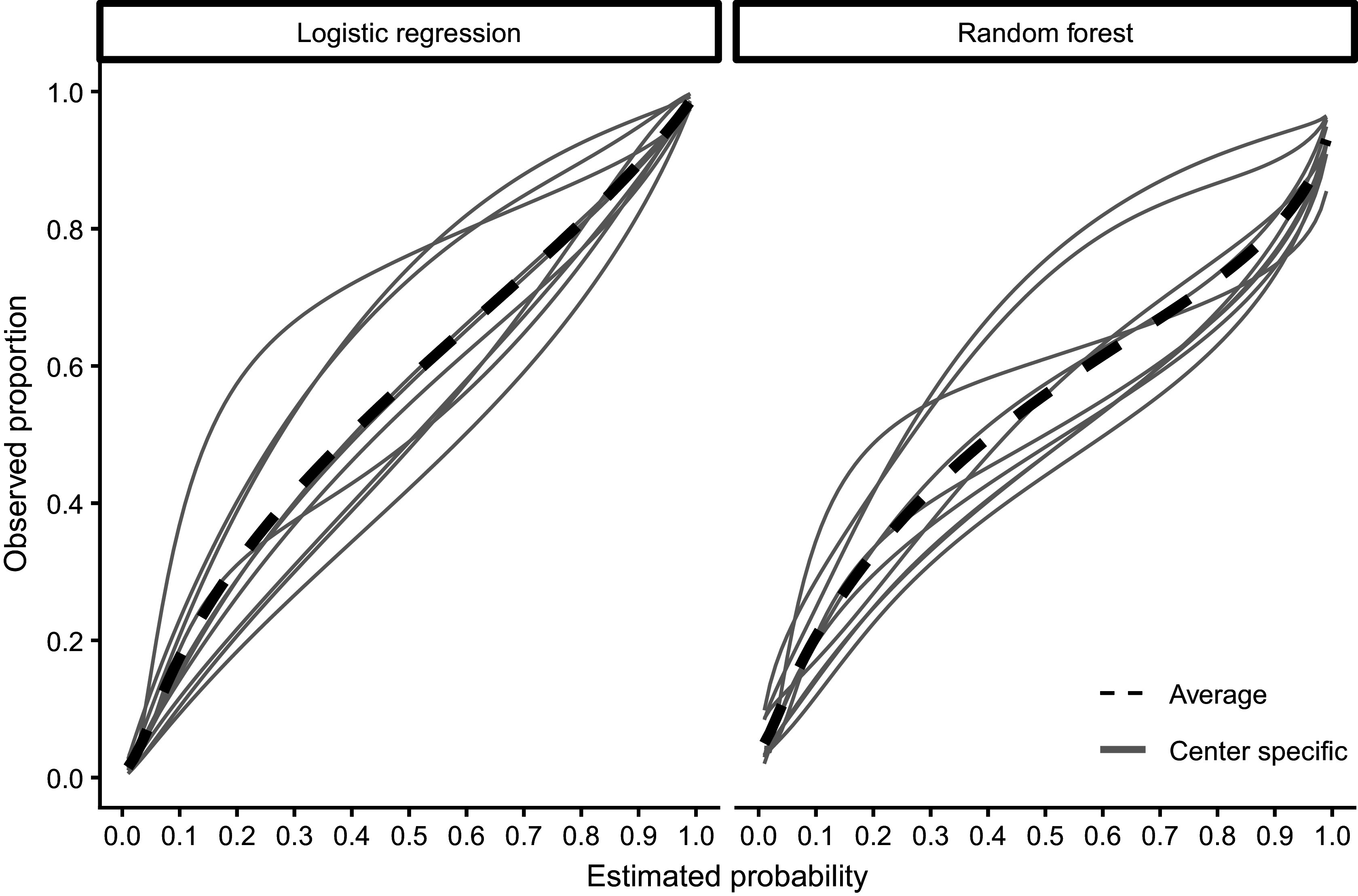
Center-specific (grey) and average true calibration plots for the synthetic data with 1000000 observations per center.

We used the synthetic data to validate the calibration of the published ADNEX model without CA125 in each center.^26^ We defined 15 scenarios based on the number of centers (2, 5, 10) and validation data EPC (20, 100, 200, 500, 1,000). We repeated this 1,000 times by randomly drawing validation datasets. If the number of centers was 2 or 5, the centers were randomly chosen as well. In each repetition, we calculated MSCE for the two true risks. True center-specific curves were obtained by training flexible calibration models (splines with five knots) on all 1 million synthetic patients from that center (Figure 6). We then compared the center-specific estimated observed proportions and the true probabilities per center in a fixed grid of 100 values. The only method that obtained center-specific curves borrowing information from other clusters is the MIX-C method, and we compare this to standard flexible calibration with LOESS and restricted cubic splines with three knots (stage 1 of 2MA-C).

### 4.2 Results

**Figure 7:**
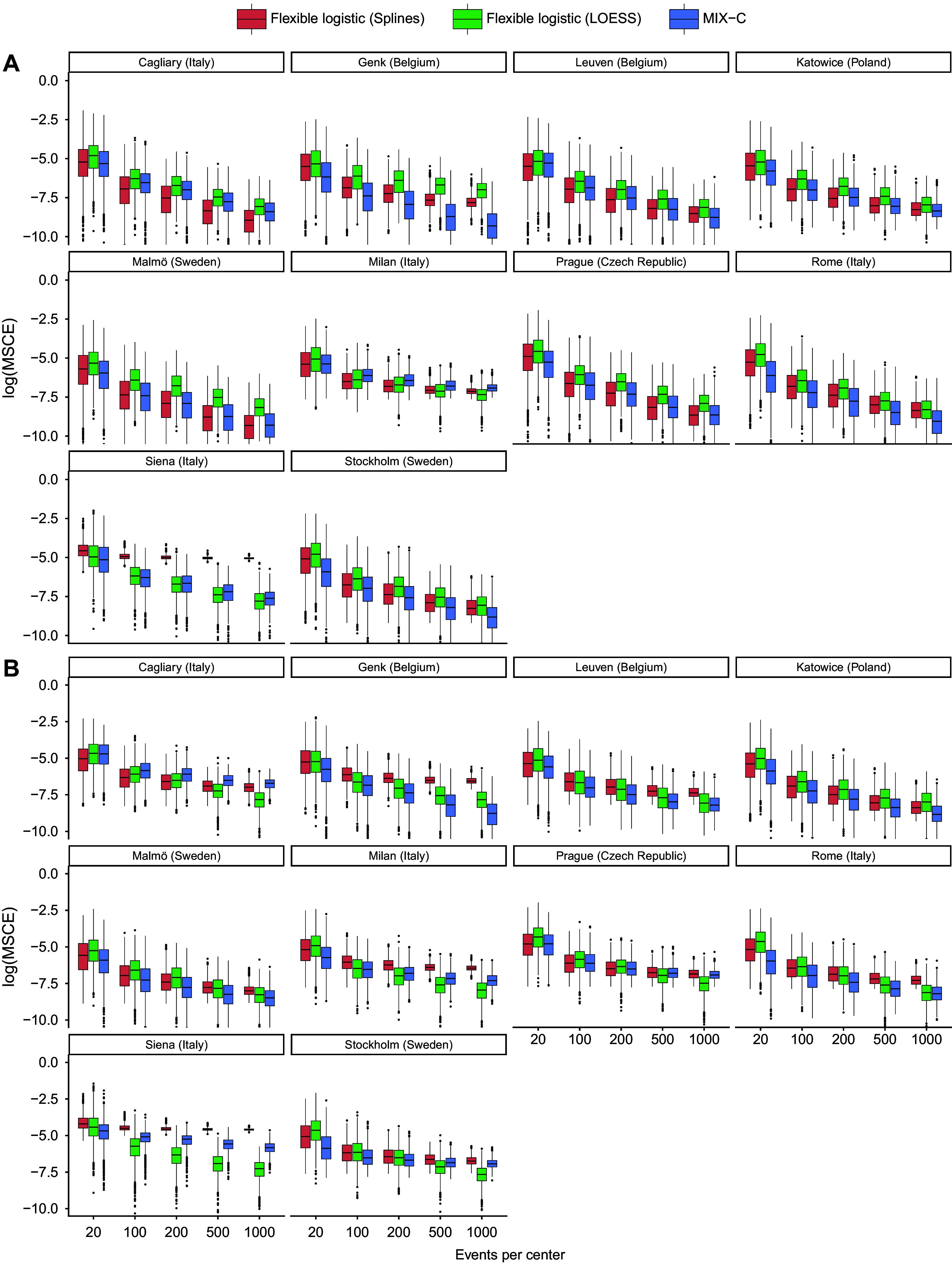
Center-specific results of log(MSCE) by the number of events per center for the logistic truth (a) and the random forest (b) truth.

Median MSCE for the synthetic data analysis with 1,000 iterations is shown in Table 4 and by center in Figure 7. MIX-C was the best-performing method in all scenarios when the truth was based on a logistic regression, with splines working equally well in five scenarios. When the truth was based on a random forest model, MIX-C was the best with small validation samples (EPC < 500) and LOESS when the sample size was above 500 events per center. Borrowing information from other clusters is an example of a bias–variance trade-off and leads to improved accuracy for the estimated cluster-specific curves for clusters with modest sample sizes. Additionally, the performance by approach varied considerably between centers (Figure 7); that is, the best approach varied by center.

**Table 4:** Median MSCE for the synthetic data study for center-specific results. MSCE is presented multiplied by 100. Bold numbers indicate best performance across approaches

		Logistic regression truth			Random forest truth		
EPC	N. of centers	Flexible logistic (splines)	Flexible logistic (LOESS)	MIX-C	Flexible logistic (splines)	Flexible logistic (LOESS)	MIX-C
20	2	0.54 (0.21–1.11)	0.66 (0.29–1.37)	**0.52 (0.23–1.01)**	0.65 (0.28–1.38)	0.81 (0.39–1.63)	**0.58 (0.25–1.19)**
20	5	0.57 (0.22–1.19)	0.68 (0.30–1.43)	**0.39 (0.15–0.85)**	0.66 (0.26–1.31)	0.83 (0.39–1.63)	**0.44 (0.19–0.96)**
20	10	0.55 (0.22–1.13)	0.67 (0.3–1.41)	**0.33 (0.13–0.72)**	0.64 (0.27–1.31)	0.81 (0.38–1.62)	**0.38 (0.16–0.85)**
100	2	**0.13 (0.06–0.29)**	0.19 (0.10–0.36)	**0.13 (0.06–0.27)**	0.19 (0.10–0.38)	0.19 (0.09–0.35)	**0.17 (0.08–0.35)**
100	5	0.13 (0.05–0.28)	0.18 (0.09–0.34)	**0.12 (0.05–0.23)**	0.19 (0.10–0.39)	0.19 (0.09–0.36)	**0.15 (0.07–0.32)**
100	10	0.13 (0.06–0.28)	0.18 (0.09–0.34)	**0.11 (0.04–0.22)**	0.20 (0.10–0.38)	0.18 (0.09–0.35)	**0.14 (0.06–0.30)**
200	2	**0.08 (0.03–0.15)**	0.11 (0.06–0.21)	**0.08 (0.03–0.15)**	0.14 (0.08–0.25)	0.12 (0.06–0.20)	**0.11 (0.05–0.23)**
200	5	**0.07 (0.03–0.16)**	0.12 (0.07–0.21)	**0.07 (0.03–0.14)**	0.14 (0.08–0.25)	0.12 (0.06–0.20)	**0.10 (0.05–0.21)**
200	10	**0.07 (0.03–0.16)**	0.12 (0.06–0.21)	**0.07 (0.03–0.13)**	0.14 (0.07–0.25)	0.11 (0.06–0.20)	**0.10 (0.04–0.20)**
500	2	**0.04 (0.02–0.08)**	0.06 (0.03–0.11)	**0.04 (0.02–0.08)**	0.11 (0.06–0.18)	**0.06 (0.03–0.11)**	0.07 (0.03–0.13)
500	5	0.04 (0.02–0.08)	0.06 (0.03–0.11)	**0.03 (0.02–0.07)**	0.11 (0.06–0.17)	**0.06 (0.03–0.11)**	0.07 (0.03–0.13)
500	10	0.04 (0.02–0.08)	0.06 (0.03–0.11)	**0.03 (0.01–0.07)**	0.11 (0.06–0.17)	**0.06 (0.03–0.11)**	0.07 (0.03–0.13)
1000	2	0.03 (0.01–0.06)	0.04 (0.02–0.06)	**0.02 (0.01–0.05)**	0.10 (0.05–0.15)	**0.04 (0.02–0.06)**	0.05 (0.02–0.11)
1000	5	0.03 (0.01–0.06)	0.04 (0.02–0.07)	**0.02 (0.01–0.04)**	0.10 (0.05–0.15)	**0.04 (0.02–0.07)**	0.06 (0.02–0.11)
1000	10	0.03 (0.01–0.06)	0.04 (0.02–0.07)	**0.02 (0.01–0.04)**	0.10 (0.05–0.15)	**0.04 (0.02–0.07)**	0.06 (0.02–0.11)

*Note:* The center-specific true curves are based on a flexible logistic model with restricted cubic splines. The lower the number, the closer the estimated center-specific calibration curve is to the true calibration curve in each cluster. Rounded to two decimals. In bold are the best working approach(es) for estimating observed proportions excluding CG-C methods.

## 5 Discussion

Calibration of CPMs is crucial since it is related to the usefulness of the recommended clinical decisions provided by the model.^3^ Evaluation of calibration performance is best done with flexible calibration plots.^4^^,^ ^20^ In this work, we introduced three methods for obtaining flexible calibration plots that account for clustering in the dataset. The first method extended the grouped calibration plot using bivariate random effects meta-analysis (CG-C method); the second method was a two-step approach in which flexible cluster-specific plots were combined through random effects meta-analysis. We generated cluster-specific plots in two ways: using restricted cubic splines (2MA-C splines) or LOESS (2MA-C LOESS). The third method used a mixed effects logistic calibration model using restricted cubic splines and random intercepts and slopes per cluster (MIX-C). Through a simulation study and a study using synthetic data from patients with an ovarian tumor, we observed that MIX-C and 2MA-C (splines) worked best to obtain the calibration plot in the cluster with the average effect (simulation study) and MIX-C for the cluster-specific calibration plots (synthetic data). The coverage probability of the PI was suboptimal for all methods and all scenarios with 2MA-C (splines) standing out as the best across scenarios. A disadvantage of 2MA-C (splines) is that the estimated calibration curves per cluster deviated more from the true curves per cluster than those obtained by MIX-C, which uses shrinkage to obtain better cluster-specific calibration curves. While CG-C may work well too to obtain a calibration plot in the cluster with the average effect when using 10 groups based on deciles, it worked poorly when using 10 groups based on equal intervals of the estimated risk. However, the grouped approach tends to depend on the number and types of groups, which is undesirable.^19^^,^ ^44^ Obviously, the other methods depend on the level of smoothing of the spline or LOESS, but we automatically selected these parameters based on statistical goodness of fit to reduce the modeler’s choices. We recommend using 2MA-C (splines) to estimate the curve with the average effect and 95% PI and MIX-C for the cluster-specific curves, especially when the sample size per cluster is limited. In our simulation, 2MA-C (LOESS) had the worst performance, possibly due to the logistic data generation mechanism. In real-world scenarios, the association of outcome and predictors tends to be more complex; therefore, a more flexible approach could be useful, as shown in the synthetic data with a non-linear data-generating mechanism and large validation sample sizes per cluster. In this work, we used the method only for external validation, but the methods also apply to model development with clustered data to explore between-cluster calibration heterogeneity during internal–external validation. We provide ready-to-use R functions to plot the curves and obtain numerical results in the OSF repository, and they are incorporated into the *CalibrationCurves* package in CRAN.^45^

Munoz et al.^46^ developed similar methodologies to derive calibration plots in large clustered datasets (preprint published after our initial submission). In their work, they presented four approaches to obtain overall calibration plots. Although their rationale is similar to ours in some cases, the implementation is different. The stacking approach is similar to what we define as flexible curve ignoring clustering (Section 2.3), where all the data from different clusters is combined into a single dataset and a calibration model is fitted. Their “one-step meta-analysis” approach corresponds to our MIX-C method where a mixed model is fitted. Their “two-step meta-analysis” method is similar to 2MA-C, but they also present a variant where instead of fitted probabilities the model parameters are aggregated. Finally, they introduced an approach where the calibration model is a generalized estimating equation. None of the methodologies include the estimation of PI. The work is illustrated with an evaluation of a model to diagnose deep vein thrombosis, but no simulation study is included to evaluate the performance of the methodologies or to compare them to our results.

The main strength of our methodologies is the inclusion of a heterogeneity measure through the PI. These intervals indicate, for every estimated risk, the range within which the observed proportion may fall in a new cluster. It is crucial to differentiate the calibration in the cluster with the average effect with the cluster-specific calibration represented in the PI. While a model can be well calibrated in the cluster with the average effect, this does not necessarily imply that the model is perfectly calibrated in every individual cluster. Statements such as “the model was well calibrated” based on the cluster with the average effect calibration plot should be avoided or at least accompanied by a clarification that this only applies to the summary curve. Using the interpretation of a calibration curve with the average effect for a specific cluster might lead to incorrect decision making. For example, the curve with average effect suggests overestimation of risks, but in some clusters, the model might be underestimating them. Although no method provided PI with correct coverage in all investigated simulation scenarios (Figure 5 and Supplementary Figure S10), they show a more realistic picture than the cluster-ignorant CI. In general, all methods underestimate the heterogeneity between clusters, therefore yielding too narrow PI, especially for estimated risks far from prevalence and when the validation sample size is small. PI of MIX-C had an odd behavior where it tended to have too narrow or too wide PI across the calibration curve. For the rest of the approaches, increasing the number of clusters in the validation when the number of events per cluster was low caused the PI to be too narrow (Supplementary Figures S12–S16). More research to fix the suboptimal performance of the PI is needed where Bayesian-based PI derivation could be a solution.^47^^–^ ^49^ Another limitation concerning CG-C and 2MA-C is that they present calibration plots based on meta-analysis of independent points. These approaches create the plot by joining each pointwise estimate of the observed proportion instead of creating a model for the whole plot and therefore are dependent on the number of points. Each of these points represents the calibration in the cluster with the average effect conditional on the estimated risks. We assume that all pointwise estimates form the calibration plot in the cluster with the average effect, but in the modeling process, each point is independent.

This study is a phase two methodological study that focused on the presentation of the methodology and assessments in a limited number of settings.^25^ Further research should therefore evaluate the methods in more extended settings and applications. For example, simulations could focus on more complex truths, such as multiple predictors with random slopes for their effects on the outcome, continuous predictors with non-linear associations with the outcome under study, and prediction models based on flexible machine learning methods such as random forests, boosting approaches, or neural networks. Instead of performing a larger simulation study, we decided to focus on synthetic data based on real data from 10 hospitals on women with an ovarian tumor with the aim of building diagnostic prediction models to estimate the risk of malignancy. These data were used to evaluate an existing prediction model, ADNEX, which is a multinomial logistic regression model using random intercepts and uniform shrinkage of model coefficients.^26^ In this synthetic data analysis, we focused on the cluster-specific performance of MIX-C by varying the validation sample. MIX-C showed good results, especially when the sample size is limited, while non-clustered approaches generally performed worse.

## Supporting information

10.1017/rsm.2025.10046.sm001 Barreñada et al. supplementary material Barreñada et al. supplementary material

## Data Availability

Data and code to reproduce results and figures are available in a public, open-access repository (https://osf.io/aj8ew/). All data relevant to the study are included in the article or uploaded as supplementary information, except the motivating example data and the synthetic data due to privacy concerns.

## Supplementary material

To view supplementary material for this article, please visit http://doi.org/10.1017/rsm.2025.10046.
