# Supplementary material for: Clustered flexible calibration plots for binary outcomes using random effects modeling
Source: Res Synth Methods. 2025 Dec 29;17(3):567–88. doi: 10.1017/rsm.2025.10046 (PMC13126218; doi:10.1017/rsm.2025.10046)
Supplement: Barreñada et al. supplementary material [file S175928792510046Xsup001.pdf]

CLUSTERED FLEXIBLE CALIBRATION PLOTS FOR BINARY OUTCOMES USING RANDOM EFFECTS MODELING

Lasai Barreñada, Bavo De Cock Campo, Laure Wynants, Ben Van Calster

SUPPLEMENTARY MATERIAL

Corresponding author

Ben Van Calster

KU Leuven, Department of Development and Regeneration

Herestraat 49 box 805

3000 Leuven

Belgium

Ben.vancalster@kuleuven.be

1 APPENDIX

A1. Clustered group calibration (CG-C)

Clustered group calibration can be seen as an extension of the traditional grouped calibration or binning calibration, where the data is split into equally sized groups based on the distribution of estimated risks, and the calibration curve shows for each group the estimated prevalence of the event on the y-axis and the mean estimated risks on the x-axis.

CG-C (grouped)

1. For each cluster $j = (1, \dots, J)$, we group the estimated probabilities into Q quantiles. Quantiles typically differ by cluster.
2. For each cluster *j* within quantile *q*, we calculate the mean outcome $\bar{y}_{qj}$, the mean predicted probability $\bar{\hat{\pi}}_{qj}$ and the number of observations n_{qj}.
3. To obtain the pooled estimated risk and observed proportion, we perform a random-effects bivariate meta-analysis using the logit-transformed $\bar{y}_{qj}$ and $\bar{\hat{\pi}}_{qj}$ for each quantile *q*. We use an unstructured covariance matrix[1–3] and estimate both the within-and between-cluster heterogeneity.

Using the fitted model, we estimate the observed proportion and estimated risk of cluster j within quantile q. The variance of the random effects (between-cluster variability) as well as the sampling error (within-cluster variability) are captured by the covariance matrices. To fit the model, we utilize the `rma.mv` function of the `metafor` package with cluster as the grouping factor and an unstructured variance-covariance matrix (see https://wviechtb.github.io/metafor/reference/rma.mv.html for a comprehensive overview).Confidence intervals are obtained with profile likelihood, and prediction intervals are calculated as explained in the `metafor` documentation.

CG-C (interval)

The algorithm is the same as CG-C (grouped) but in step 1, instead of grouping based on quantiles, we create Q intervals evenly dividing the probability space (0 – 1). Then steps 2 and 3 are identical.

A2. Two stage meta-analysis (2MA-C)

The two-stage meta-analysis approach combines individual cluster specific calibration models to obtain the calibration in the cluster with the average effect. The process has two stages, first obtaining the individual cluster's calibration and then combining them using random effects meta-analysis as follows:

Stage 1

Fit a flexible calibration model (LOESS or splines, see section 2.3 of the main paper) per cluster and estimate the observed proportion for a grid of values (e.g. 100 values from 0.01 to 0.99).

We estimate the corresponding observed proportion with the calibration model over a grid ($\boldsymbol{G} = g \in \mathbb{R} \mid 0.01 \leq g \leq 0.99$).

Stage 2

Pool the observed proportion per grid value (g) using a random effects model:

$$logit({}_{s}\widehat{\pi}_{gj}) = {}_{\widehat{\pi}}\mu_g + \nu_{gj} + \epsilon_{gj}, \quad \epsilon_{gj} \sim N(0, {}_{\widehat{\pi}}\sigma^2_{gj}), \qquad \nu_{gj} \sim N(0, {}_{\widehat{\pi}}\tau^2_g)$$

With $logit({}_{s}\widehat{\pi}_{gj})$ the logit-transformed predicted probability for point g within cluster j, ν_{gj} the random effect of cluster j and ϵ_{gj} the error term. The summary estimate is obtained using inverse variance weighting

$$logit({}_{s}\widehat{\pi}_{g}) = \frac{\sum_{j=1}^{J} logit({}_{s}\widehat{\pi}_{gj})\, w_{gj}}{\sum_{j=1}^{J} w_{gj}}$$

where w_{gj} denote the weights calculated as

$$w_{gj} = \frac{1}{{}_{\widehat{\pi}}\tau^2_g + {}_{\widehat{\pi}}\sigma_{gj}}.$$

${}_{\widehat{\pi}}\tau^2_g$ is the between-cluster variability or heterogeneity estimated with REML (see Veroniki et al. for an overview of methods to estimate ${}_{\widehat{\pi}}\tau^2_g$ [4]) for point g and ${}_{\widehat{\pi}}\sigma_{gj}$ is the within-cluster standard error of point g in cluster j. We then use the fitted model per grid value to estimate the observed proportion associated with the grid value and plot the calibration curve.

The confidence interval can be calculated using the Hartung-Knapp-Sidik-Jonkman approach, which is recommended when the number of studies is small [5,6] or with the default method.

Finally we get prediction interval based on the t-distribution as explained in Higgins et al (2009) $logit({}_{s}\widehat{\pi}_{gj}) \mp t_{J-2}\sqrt{{}_{\widehat{\pi}}\tau^2_g + SE\left(logit({}_{s}\widehat{\pi}_{gj})\right)^2}$ [7] (default) where t_{J-2} denotes the t-Student distribution with *J-2* degrees of freedom or any of the supported methods for `meta` package, namely Harktung-Knapp, Kenward Roger [8], bootstrap approach[9] or based on standard normal quantile [10].

A3. One step mixed model (MIX-C)

In this approach, we estimate the observed proportion as

$${}_{o}\hat{p}_{ij} = \text{logit}^{-1}\left\{\hat{s}\left(\text{logit}\left(\widehat{\pi}(x_{ij})\right)\right) + \widehat{s_j}\left(\text{logit}\left(\widehat{\pi}(x_{ij})\right)\right)\right\}$$

where $\hat{s}$ and $\widehat{s_j}$ denote the estimated smooth effects. We take the variance of both the fixed and random components into account when calculating the variance of the linear predictor and we approximate the standard error of ${}_{o}\hat{p}_{ij}$ using the delta method. To keep the confidence interval within [0, 1], we construct the interval as

$$min\left(1, max\left(0, {}_{o}\hat{p}_{ij} \mp z_{1-\frac{\alpha}{2}} se({}_{o}\hat{p}_{ij})\right)\right)$$

where $z_{1-\frac{\alpha}{2}}$ denotes the quantile of the standard normal distribution that corresponds to the cumulative probability of $1-\frac{\alpha}{2}$ (i.e. 1.96 for a 95% CI). Prediction intervals are calculated using the *predictInterval* function in R with 10 000 samples (simulation based). This function takes into account the uncertainty at observation level (residual variance), in the fixed coefficients and in the random

effects. In this method we first obtain the random and fixed effects, then we generate n samples (default = 10000) based on a multivariate normal distribution of the random and fixed effects, separately. Then we calculate the linear predictor in each sample and predict the upper and lower limits of the prediction interval.

A4. Figures

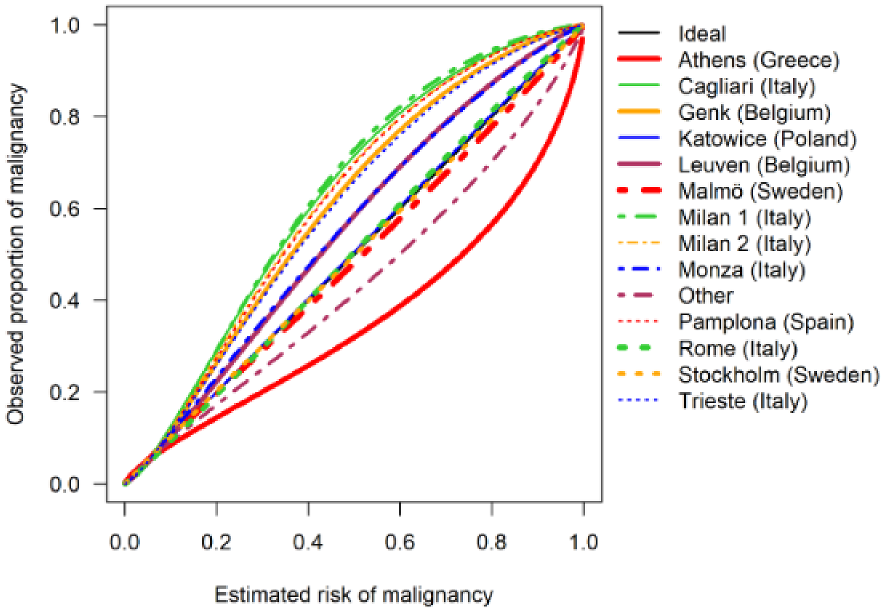

Figure S1. ADNEX without CA125 center specific logistic calibration curves in IOTA 5 dataset. Reproduced with permission from Van Calster et al (2020).[11] Copyright BMJ Publishing Group.

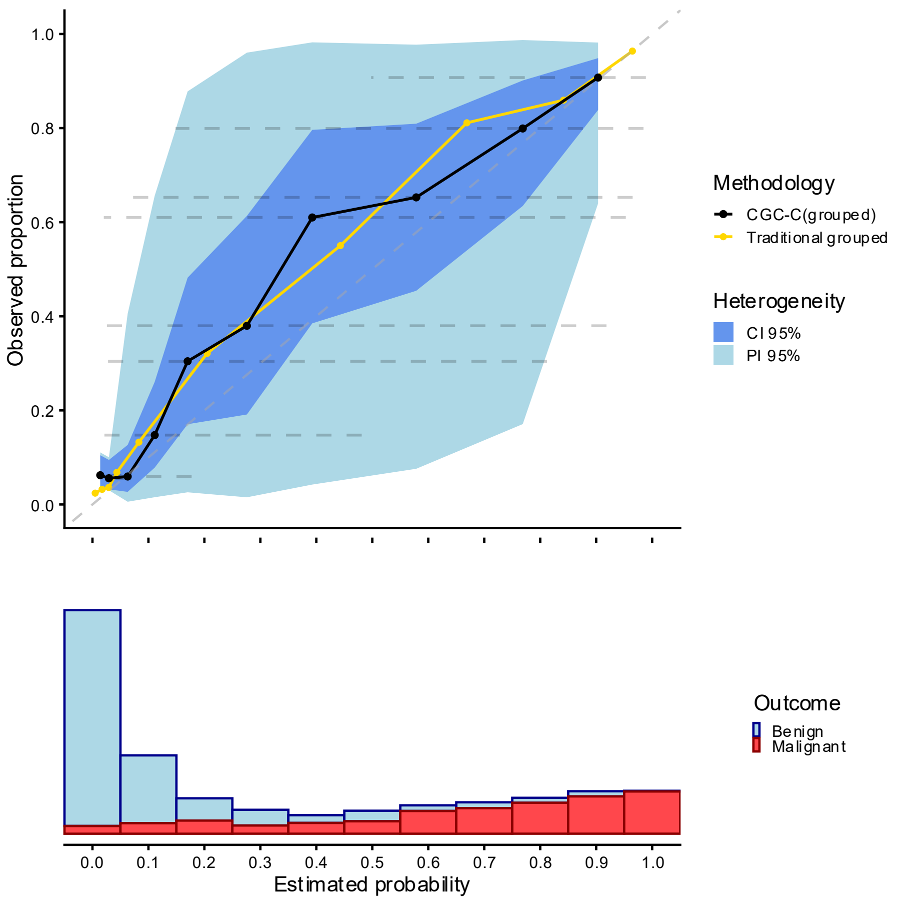

Figure S2. CG-C (grouped) and traditional grouped calibration plot with 10 quantiles and histogram of estimated risks for the ADNEX model in the motivating example. Dashed horizontal line indicates meta-analysis prediction interval across average estimated probabilities per group.

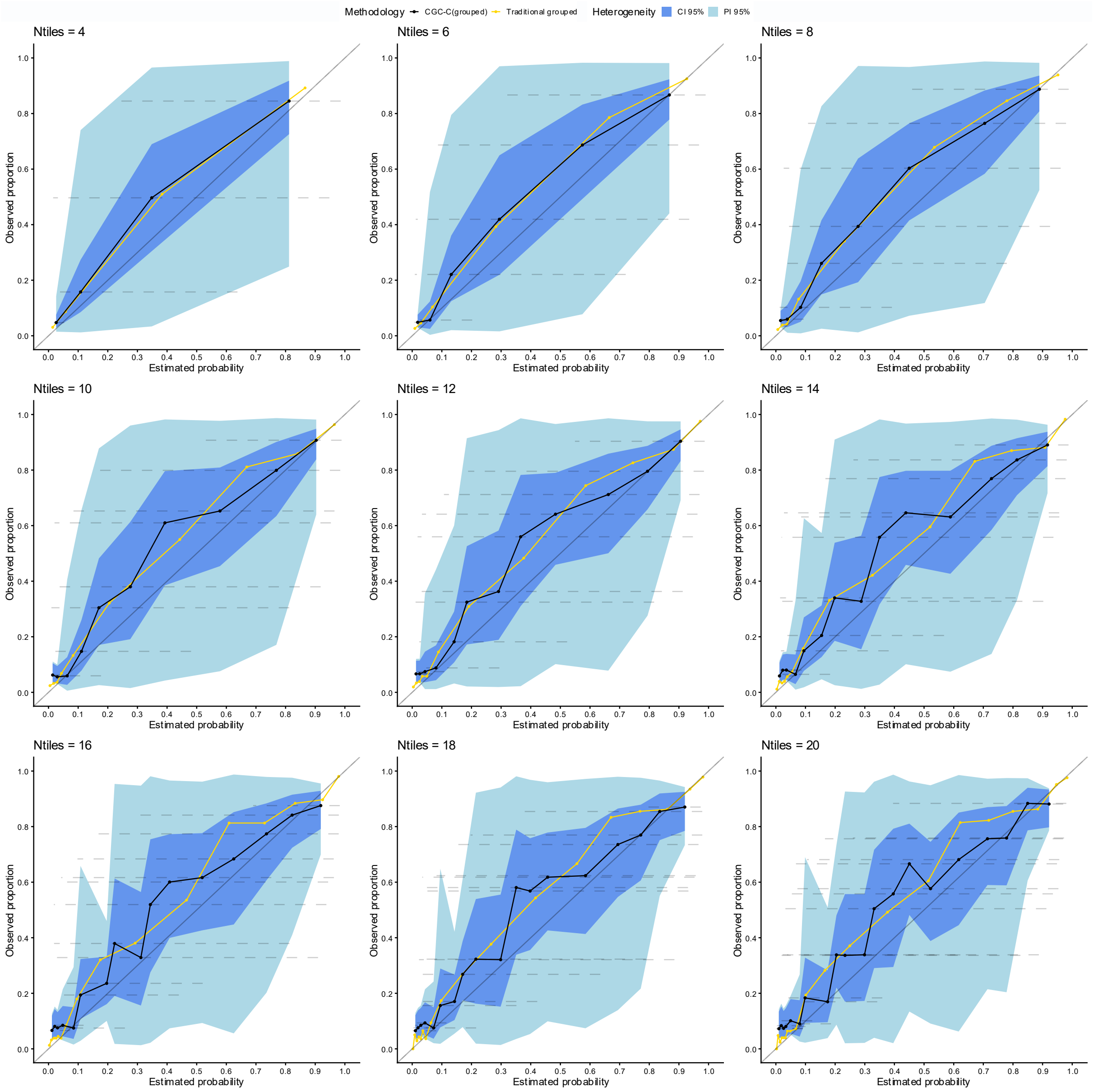

Figure S3. CG-C (grouped) and traditional grouped calibration plot with varying quantiles from 2 to 20 for the ADNEX model in the motivating example. Dashed horizontal line indicates meta-analysis prediction interval across average estimated probabilities per group.

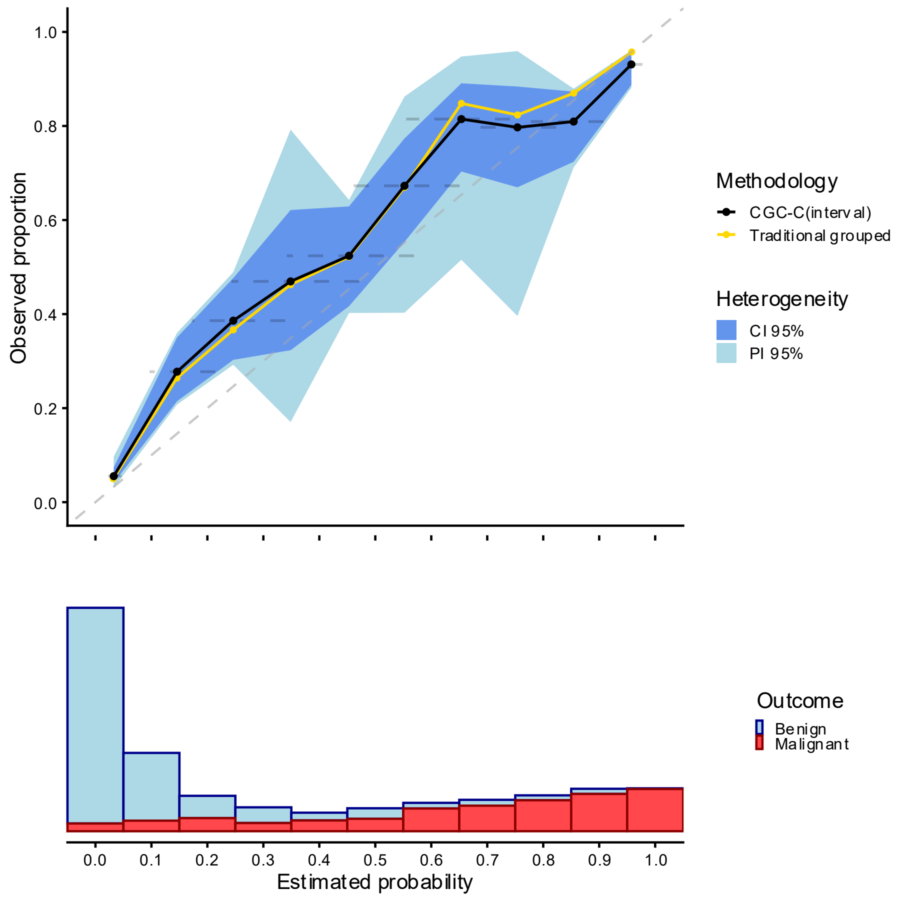

Figure S4. CG-C (interval) and traditional grouped calibration plot with 10 quantiles and histogram of estimated risks for the ADNEX model in the motivating example. Dashed horizontal line indicates meta-analysis prediction interval across average estimated probabilities per group.

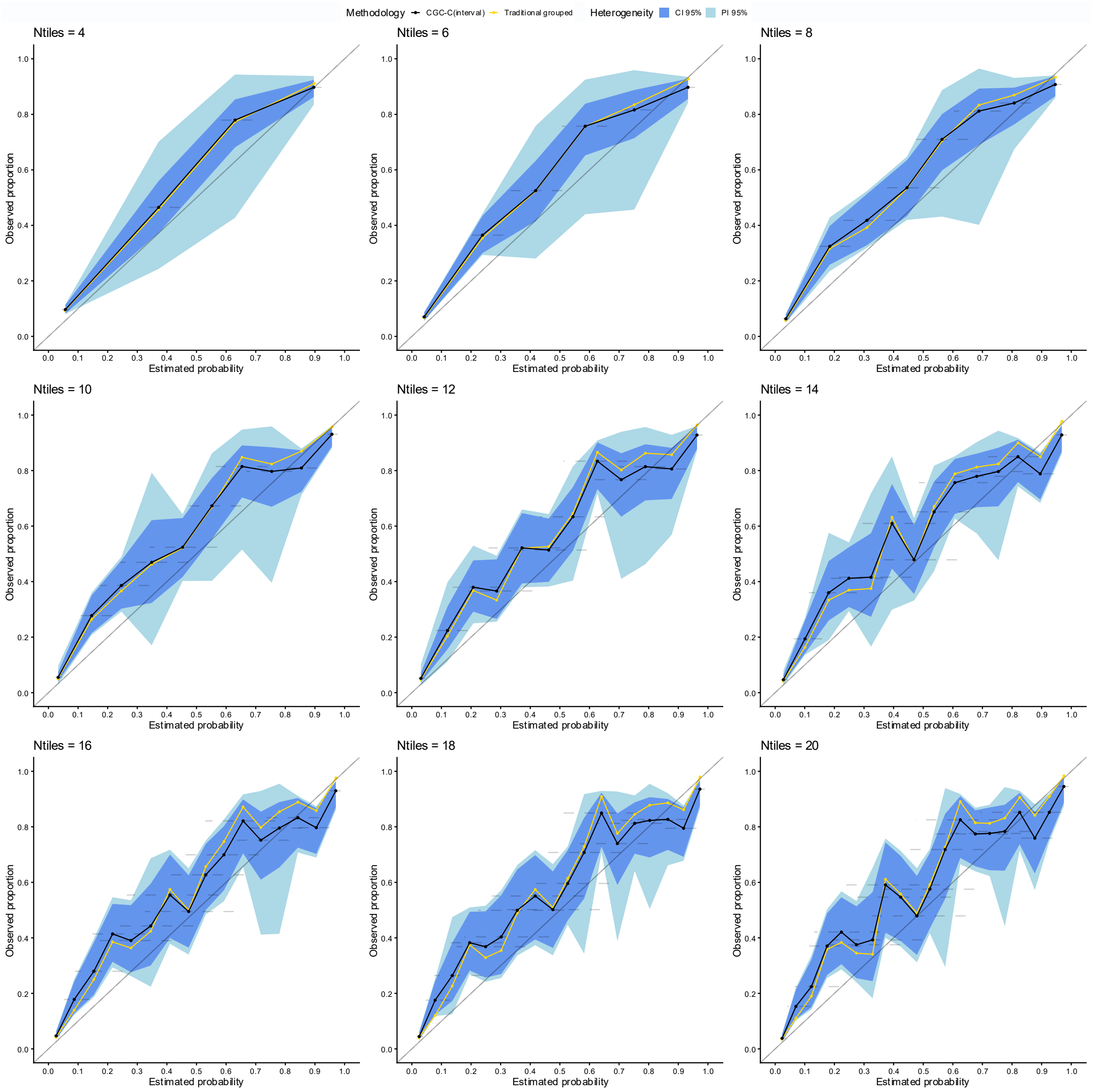

Figure S5. CG-C (interval) center-specific and traditional grouped calibration plot with varying quantiles from 2 to 20 for the ADNEX model in the motivating example.- Dashed horizontal line indicates meta-analysis prediction interval across average estimated probabilities per group.

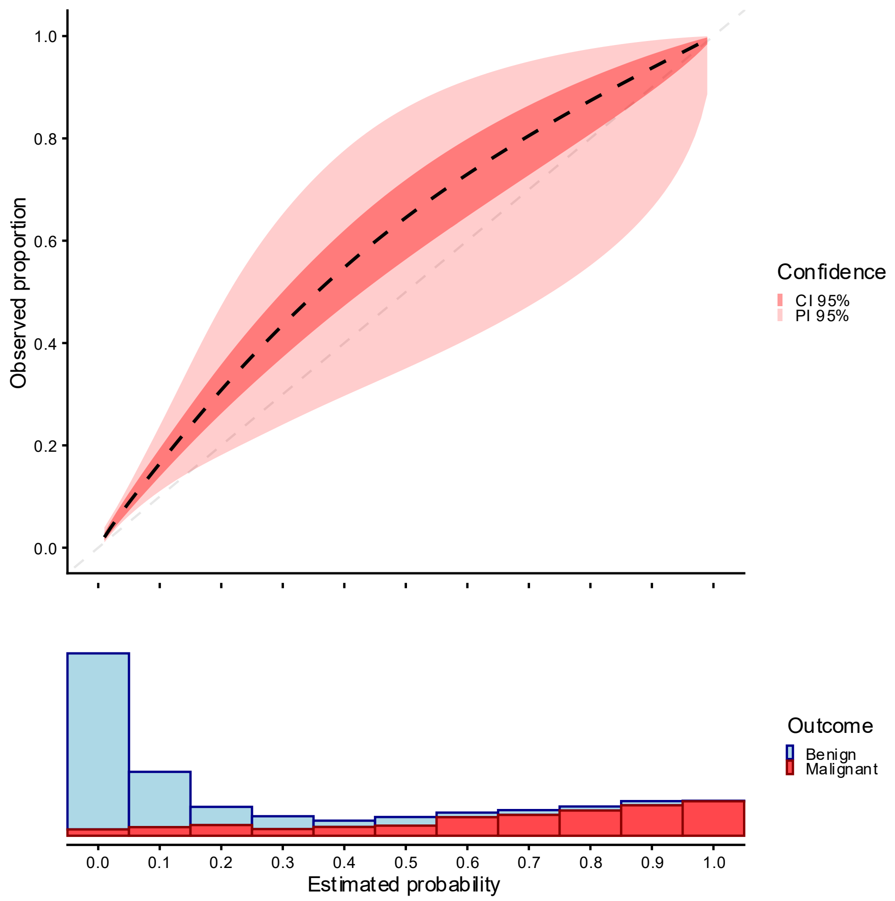

Figure S6. 2MA-C (splines) calibration plot for the ADNEX model in the motivating example with 100 grid points.

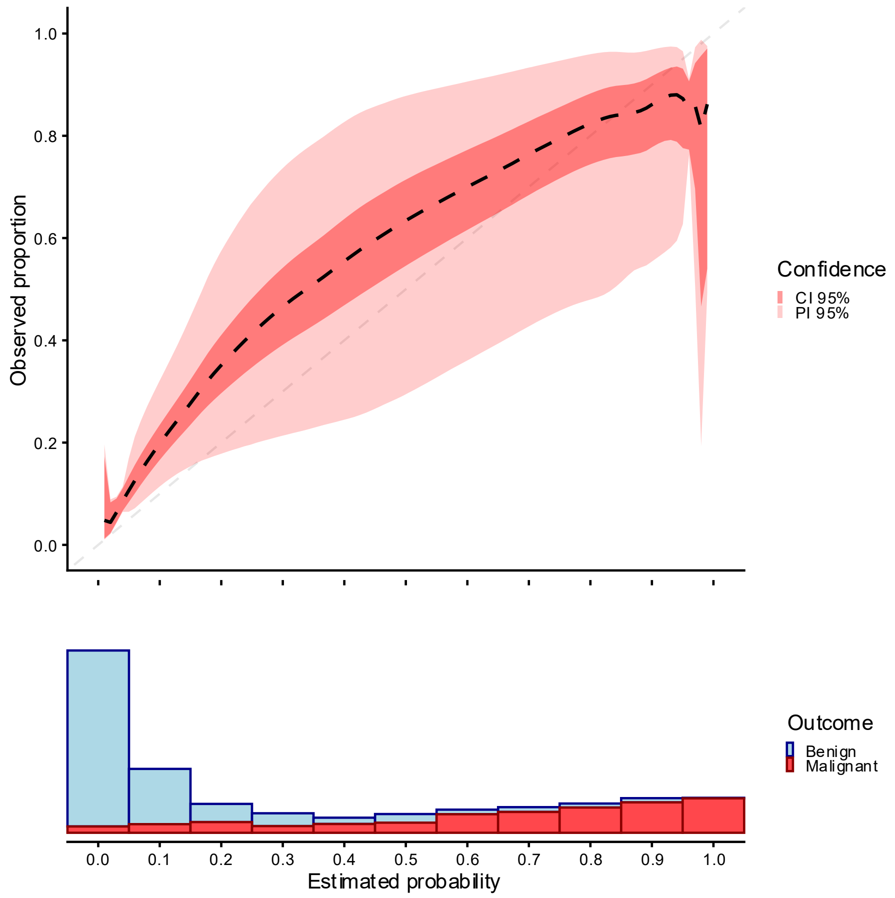

Figure S7. 2MA-C (loess) calibration plot for the ADNEX model in the motivating example with 100 grid points.

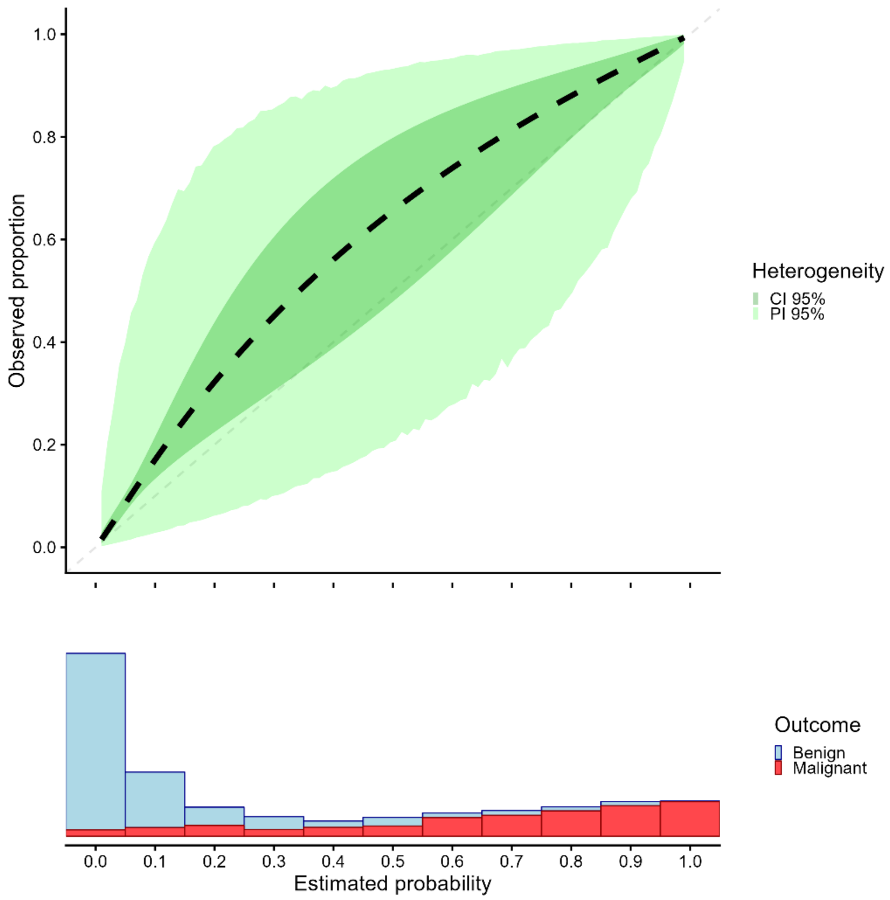

Figure S8. MIX-C calibration curve based on a model with random intercept and slopes per center and restricted cubic splines for the ADNEX model in the motivating example.

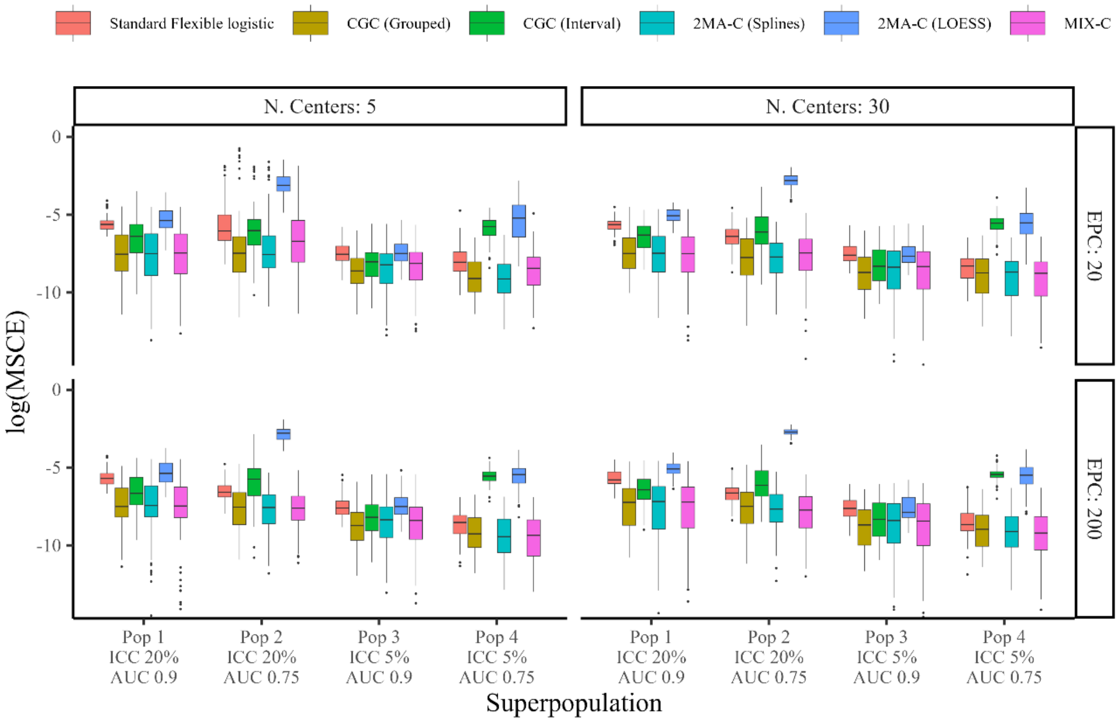

Figure S9. Boxplots of mean squared calibration error (log) for the prediction model with varying training sample size and fixed validation size of 100.000 patients in 30 centers.

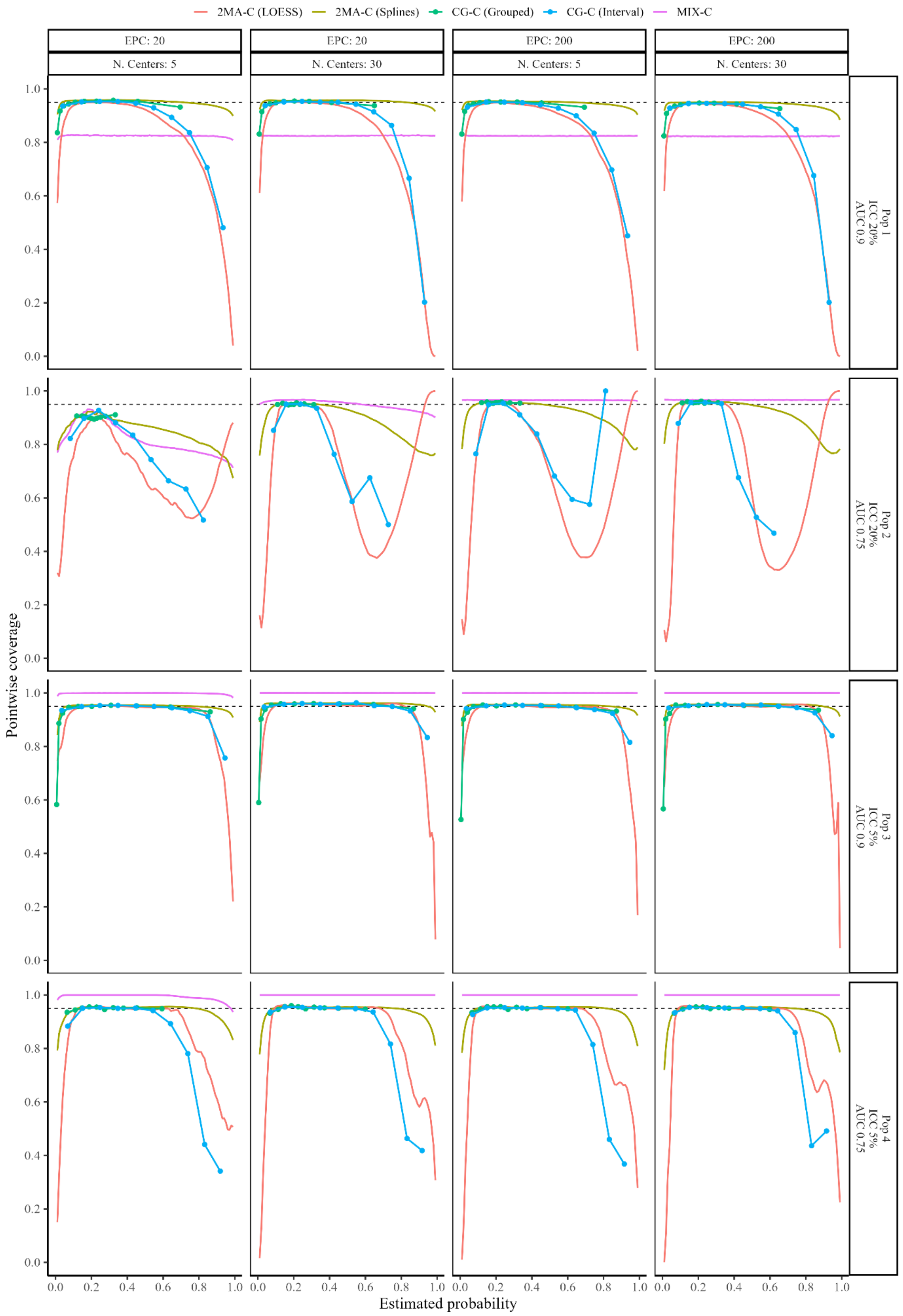

Figure S10. Pointwise prediction interval coverage across the 16 scenarios with varying training sample size and fixed validation size of 100.000 patients in 30 centers. Black dotted line indicates nominal coverage (95%).

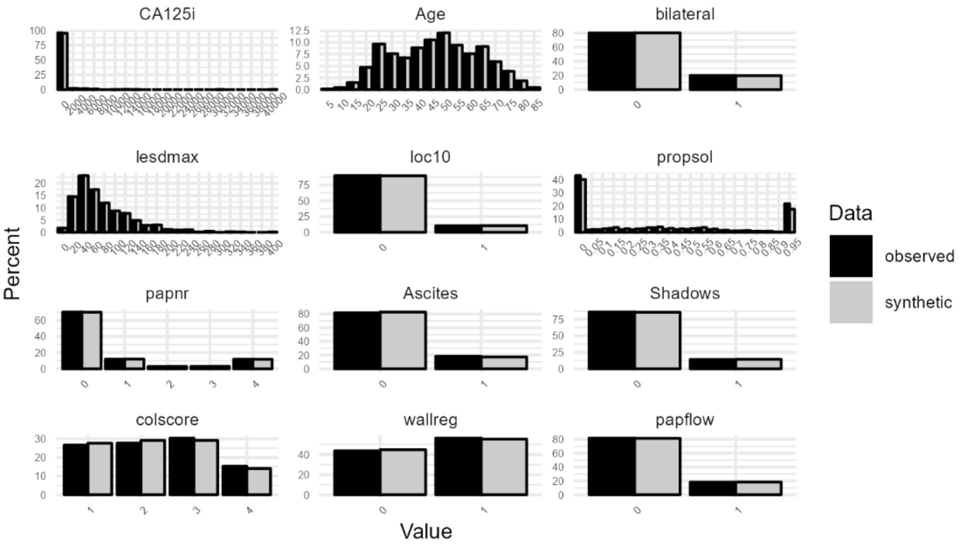

Figure S11. Quality of synthetic data generation for one of the centers (Leuven).

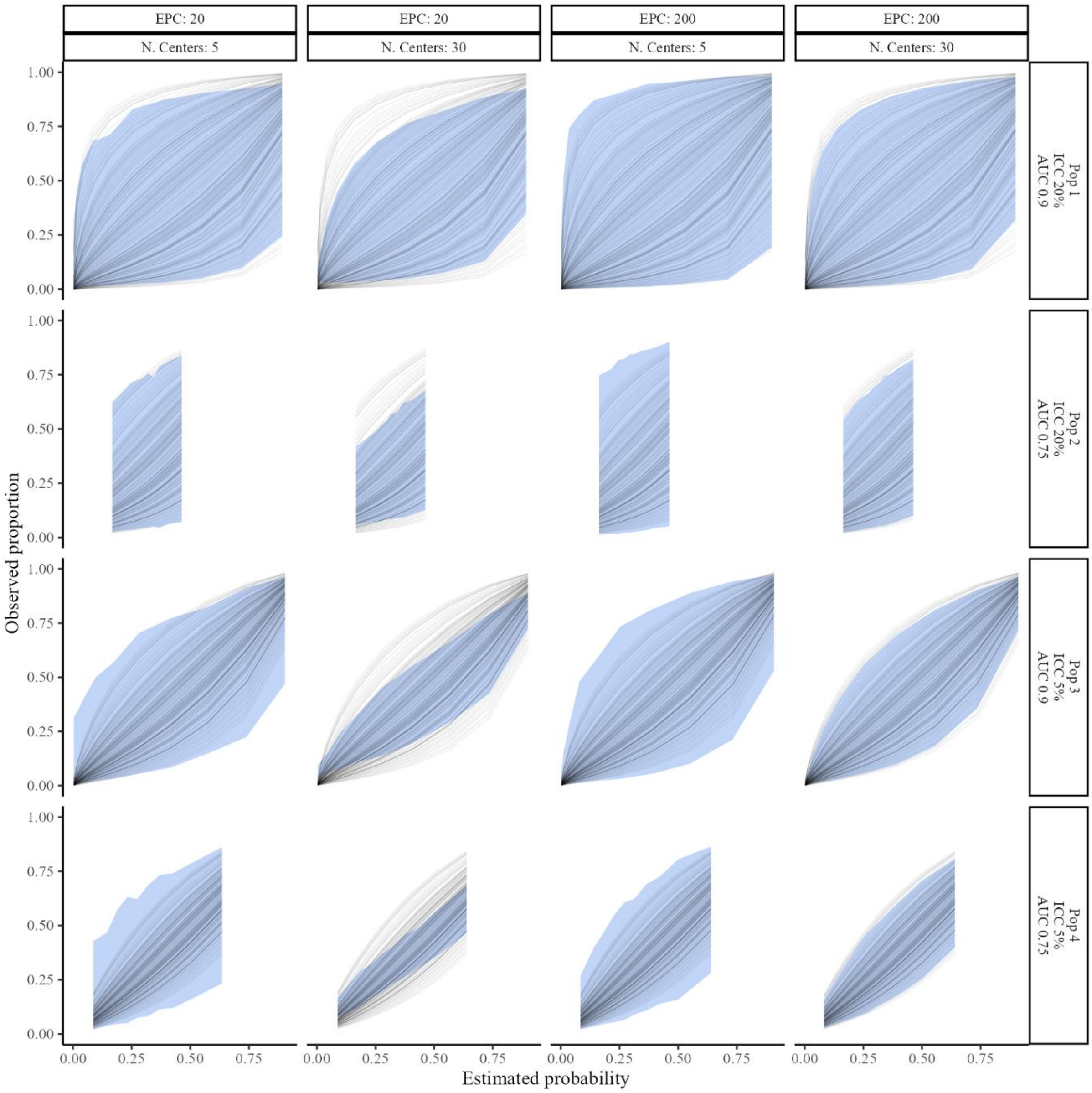

Figure S12. Average prediction interval across the different scenarios with different validation sample size for the CG-C (grouped) approach. Prediction interval is calculated as the average of the 100 iterations. Black lines represent the true center specific curves in the 200 centers of the superpopulation.

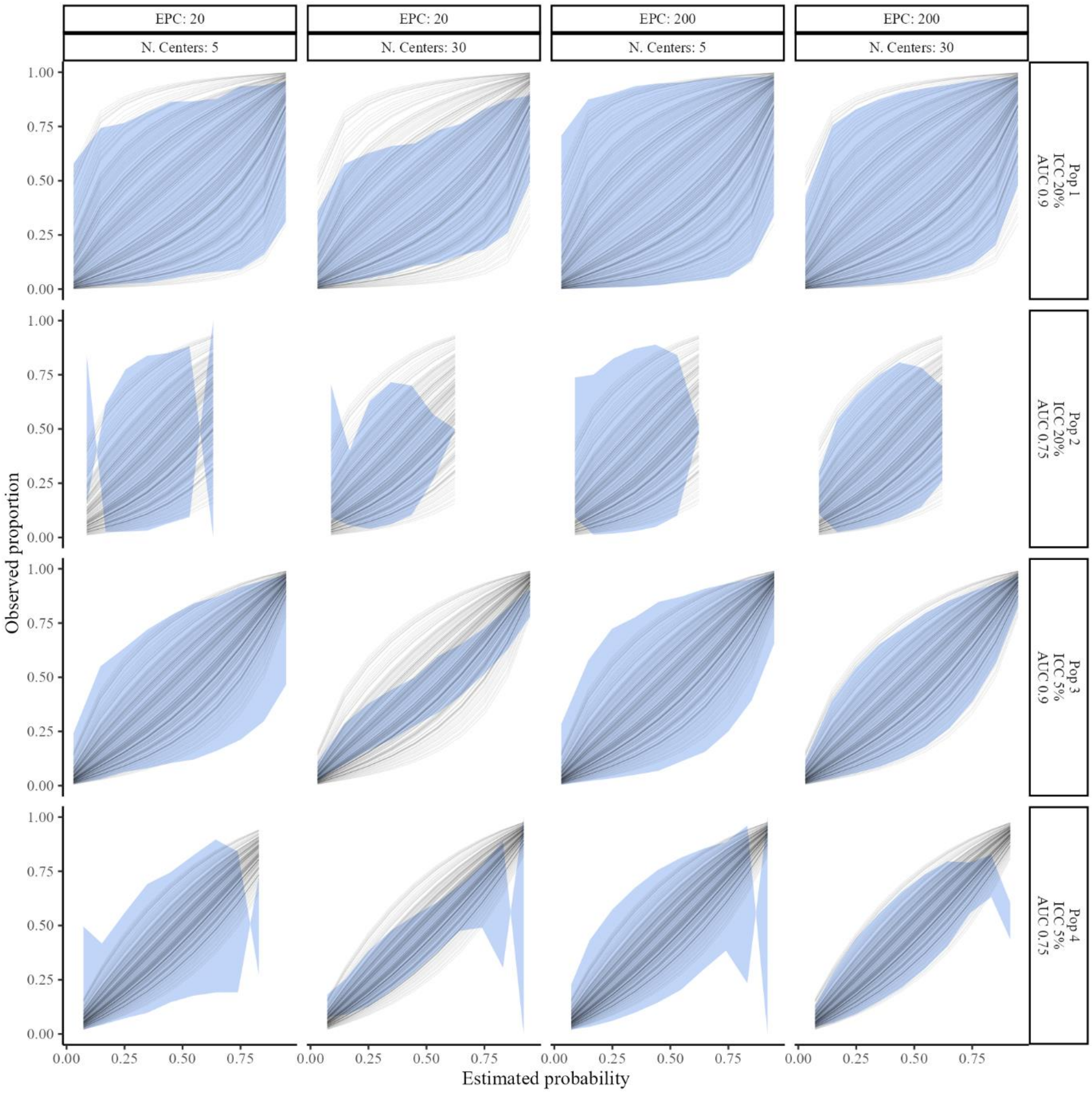

Figure S13. Average prediction interval across the different scenarios with different validation sample size for the CG-C (interval) approach. Prediction interval is calculated as the average of the 100 iterations. Black lines represent the true center specific curves in the 200 centers of the superpopulation.

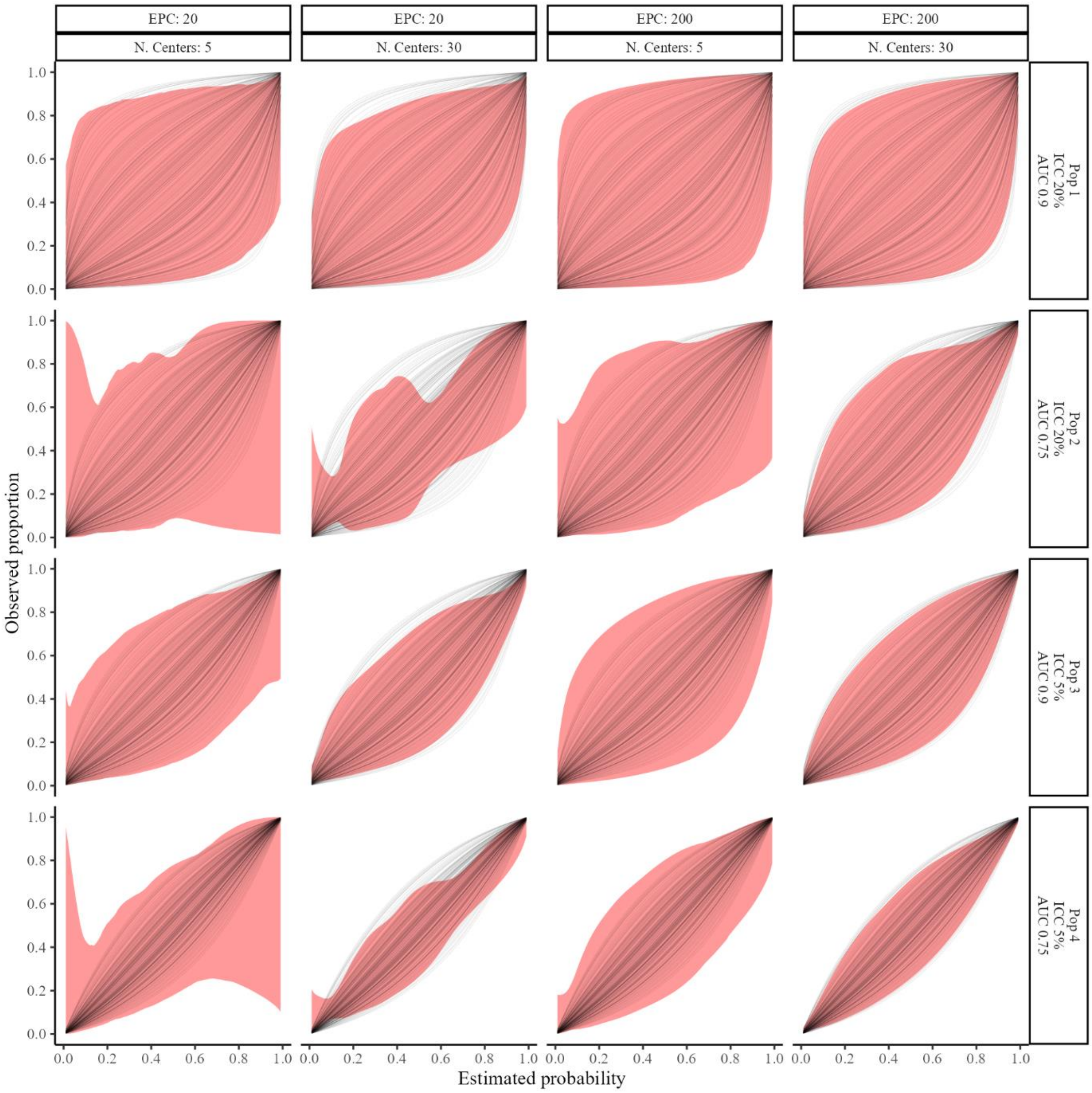

Figure S14. Average prediction interval across the different scenarios with different validation sample size for the 2MA-C (splines) approach. Prediction interval is calculated as the average of the 100 iterations. Black lines represent the true center specific curves in the 200 centers of the superpopulation.

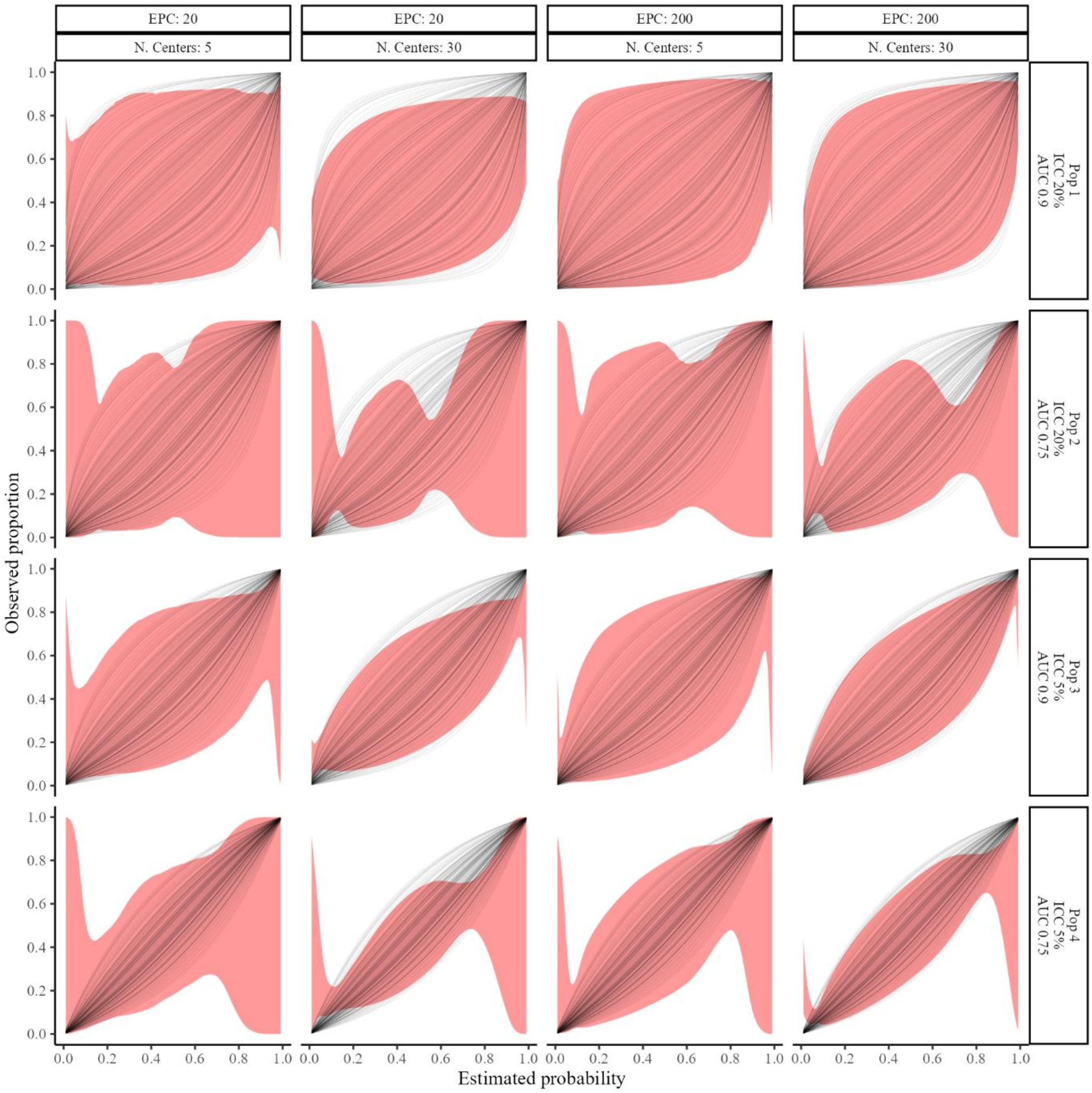

Figure S15. Average prediction interval across the different scenarios with different validation sample size for the 2MA-C (LOESS) approach. Prediction interval is calculated as the average of the 100 iterations. Black lines represent the true center specific curves in the 200 centers of the superpopulation.

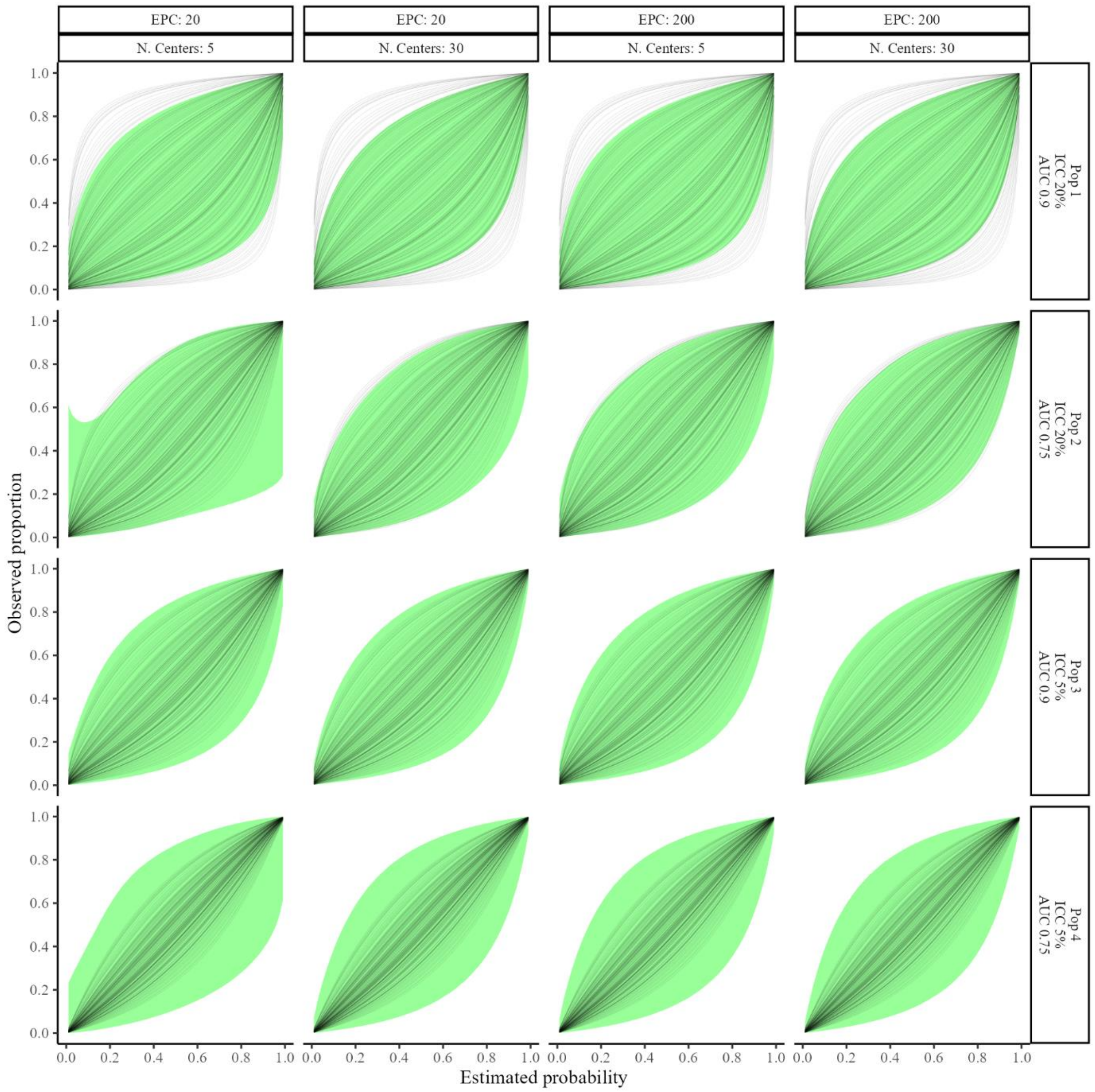

Figure S16. Average prediction interval across the different scenarios with different validation sample size for the MIX-C approach. Prediction interval is calculated as the average of the 100 iterations. Black lines represent the true center specific curves in the 200 centers of the superpopulation.

A5. Tables

Superpopulation	AUC	ICC	Event rate (range)	Formula
P1	0.9	0.2	0.3 (0.04-0.74)	$\pi(\boldsymbol{x}_{ij}) = -1.6054 - 2.09062x_{ij} + u_j$<br>$u_j \sim N(0{,}1.559)$
P2	0.75	0.2	0.3 (0.05-0.75)	$\pi(\boldsymbol{x}_{ij}) = -1.0122 + 0.4199x_{ij} + u_j$<br>$u_j \sim N(0{,}1.0024)$
P3	0.9	0.05	0.3 (0.14-0.51)	$\pi(\boldsymbol{x}_{ij}) = -1.5943 + 2.3875x_{ij} + u_j$<br>$u_j \sim N(0{,}0.7827)$
P4	0.75	0.05	0.3 (0.14-0.52)	$\pi(\boldsymbol{x}_{ij}) = -1.0244 - 0.9273x_{ij} + u_j$<br>$u_j \sim N(0{,}0.5183)$

Table S1. Superpopulation characteristics

References

1. van Houwelingen HC, Arends LR, Stijnen T. Advanced methods in meta-analysis: multivariate approach and meta-regression. *Stat Med*. 2002;21(4):589-624. doi:10.1002/sim.1040

2. Berkey CS, Hoaglin DC, Mosteller F, Colditz GA. A random-effects regression model for meta-analysis. *Stat Med*. 1995;14(4):395-411. doi:10.1002/sim.4780140406

3. Reitsma JB, Glas AS, Rutjes AWS, Scholten RJPM, Bossuyt PM, Zwinderman AH. Bivariate analysis of sensitivity and specificity produces informative summary measures in diagnostic reviews. *J Clin Epidemiol*. 2005;58(10):982-990. doi:10.1016/j.jclinepi.2005.02.022

4. Veroniki AA, Jackson D, Viechtbauer W, et al. Methods to estimate the between-study variance and its uncertainty in meta-analysis. *Res Synth Methods*. 2016;7(1):55-79. doi:10.1002/jrsm.1164

5. Schwarzer G, Carpenter JR, Rücker G. *Meta-Analysis with R*. Cham: Springer International Publishing; 2015. doi:10.1007/978-3-319-21416-0

6. Snell KI, Ensor J, Debray TP, Moons KG, Riley RD. Meta-analysis of prediction model performance across multiple studies: Which scale helps ensure between-study normality for the C-statistic and calibration measures? *Stat Methods Med Res*. 2018;27(11):3505-3522. doi:10.1177/0962280217705678

7. Higgins JPT, Thompson SG, Spiegelhalter DJ. A re-evaluation of random-effects meta-analysis. *J R Stat Soc Ser A Stat Soc*. 2009;172(1):137-159. doi:10.1111/j.1467-985X.2008.00552.x

8. Partlett C, Riley RD. Random effects meta-analysis: Coverage performance of 95% confidence and prediction intervals following REML estimation. *Stat Med*. 2017;36(2):301-317. doi:10.1002/sim.7140

9. Nagashima K, Noma H, Furukawa TA. Prediction intervals for random-effects meta-analysis: A confidence distribution approach. *Stat Methods Med Res*. 2019;28(6):1689-1702. doi:10.1177/0962280218773520

10. Skipka G. The inclusion of the estimated inter-study variation into forest plots for random effects meta-analyses – a suggestion for a graphical representation. https://abstracts.cochrane.org/2006-dublin/inclusion-estimated-inter-study-variation-forest-plots-random-effects-meta-analyses. Published 2006.

11. Van Calster B, Valentin L, Froyman W, et al. Validation of models to diagnose ovarian cancer in patients managed surgically or conservatively: multicentre cohort study. *BMJ*. 2020;370:m2614. doi:10.1136/bmj.m2614
